# Piezofluorochromism in Aramid Dyads: Pressure‐Triggered Luminescence Enhancement with Predictable Emission Shifts

**DOI:** 10.1002/advs.202518246

**Published:** 2025-11-05

**Authors:** Yayun Wang, Zhe Jia, Yaru Wang, Lin Wei, Zimin Hao, Yanan Wang, Jingwen Guo, Aisen Li, Lei Li, Kai Wang, Qian Li

**Affiliations:** ^1^ Key Laboratory of Quantum Materials Under Extreme Conditions in Shandong Province School of Physics Science and Information Technology Liaocheng University Liaocheng Shandong 252000 China; ^2^ Shandong Key Laboratory of Applied Technology for Protein and Peptide Drugs School of Pharmaceutical Sciences and Food Engineering Liaocheng University Liaocheng Shandong 252000 China

**Keywords:** aggregation‐induced emission, diamond anvil cell, high pressure, piezochromic luminescence, pressure‐induced emission enhancement

## Abstract

Aggregation‐induced emission has highlighted aramids as promising platforms for developing solid‐state luminophores, broadening the conventional design landscape for these materials. Nonetheless, the simultaneous achievement of high efficiency and precisely tailored fluorescence in aramids remains a considerable challenge. Herein, high‐pressure technique is employed to elucidate the critical structural determinants governing the emission intensity and shifts of aramids. Two representative aramid isomers, PP and OO, which exhibit distinct noncovalent conformation lock (NCL) effects, yield markedly different luminescence behaviors. For the PP crystal lacking NCL, pressure‐induced molecular rigidity and planarity effectively restrict intramolecular phenyl‐ring rotation. This constraint suppresses excited‐state geometric distortion and enhances the population of excited electrons in higher vibrational states, ultimately giving rise to an unusual hypsochromic shift in luminescence. In contrast, the OO crystal, stabilized by NCL, exhibits a conventional bathochromic shift in emission, attributable to structural contraction and planarization upon compression. Collectively, these findings identify intrinsic intramolecular rotation as a decisive factor in governing the piezofluorochromic behavior of aramids, and further suggest conjugation modulation as a viable strategy for the precise tailoring of their piezofluorochromic properties.

## Graphical abstract

High‐pressure investigations uncover the structural determinants of luminescence intensity and shifts in aramids, establishing intrinsic intramolecular rotation as a reliable predictor of their piezofluorochromic behavior. Leveraging this insight, conjugation modulation is proposed as a precise strategy to tune their piezofluorochromic properties, offering a rational pathway for designing pressure‐responsive organic luminophores.

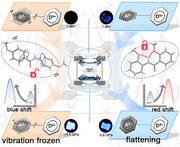

## 1 Introduction

Organic luminophores hold immense potential for diverse applications ranging from bioimaging to display lighting.^[^ ^1^, ^2^ ^]^ Nevertheless, conventional organic emitters often suffer from detrimental aggregation effects, such as π–π stacking and excimer formation, that severely quench their luminescence in the solid state.^[^ ^3^ ^]^ In such systems, the photophysical processes are governed not only by the intrinsic properties of individual molecules, but also by intermolecular packing and interactions.^[^ ^4^ ^]^ Hence, rational molecular design is essential to achieve efficient solid‐state emission. Notably, a major breakthrough in this field has been the advent of aggregation‐induced emission (AIE) materials, which are weakly emissive in dilute solution but exhibit intense luminescence upon aggregation.^[^ ^5^, ^6^ ^]^ Since its identification, AIE has greatly advanced the development of solid‐state emitters. The enhanced emission efficiency is generally ascribed to the suppression of nonradiative decay pathways, primarily resulting from the restriction of intramolecular motions in the solid state. Despite their success, most AIE luminogens rely on highly twisted conformations, such as tetraphenylethylene and its derivatives.^[^ ^7^, ^8^ ^]^ Expanding the design landscape beyond these archetypes is therefore crucial for the continued advancement of organic luminescent materials. Meanwhile, engineering aggregated luminophores that combine high efficiency with targeted fluorescence characteristics remains a significant challenge, requiring both precise structural control and a deep understanding of the relationship between molecular architecture and solid‐state photophysics.

Aromatic amides (aramids) represent a fundamental structural motif widely found in bioactive compounds and pharmaceutical agents.^[^ ^9^ ^]^ The stable and well‐defined conformations make them indispensable as chemical foldamers and scaffolds for constructing nitrogen‐containing heterocyclic and carbocyclic systems.^[^ ^10^ ^]^ In optoelectronic applications, aramids also demonstrate various attractive features, including extended π‐conjugation, versatile chemical modifiability, and structural rigidity. Historically, however, their intrinsic luminescence has received limited attention in traditional research, primarily due to certain photophysical processes, such as proton transfer, that tend to suppress emission efficiency at the molecular level.^[^ ^11^ ^]^ Recent advances in AIE have inspired renewed interest in aramids, revealing their potential as promising platforms for solid‐state luminophores.^[^ ^12^ ^]^ Importantly, a distinctive feature of aramids is the resonance between the nitrogen lone pair and carbonyl π* orbital, which imparts a high rotational barrier, excellent chemical inertness, and planar conformation of the amide bond.^[^ ^13^ ^]^ Disrupting this planarity, typically through geometric distortion, could significantly alter their physicochemical and photophysical properties.^[^ ^14^ ^]^ As such, mechanical twisting of the amides emerges as a feasible strategy for tuning their intrinsic luminescence without chemical modification. Nevertheless, achieving such modulation in a controllable manner remains rare, and the structural parameters that dictate emission efficiency and excited‐state behavior are not yet fully understood.

High pressure has recently gained attention as a precise, non‐destructive method to accurately modulate structural parameters without altering chemical composition.^[^ ^15^, ^16^ ^]^ Using a diamond anvil cell (DAC), both structural and optical properties can be monitored continuously and *in*‐*situ* under compression. This technique not only unveils the intrinsic characteristics of materials, but also helps elucidate the underlying structure‐property relationships at the atomic level.^[^ ^17^, ^18^ ^]^ In typical AIE systems, the primary effect of compression is to shorten intermolecular distances. This effect could enhance the polarization between adjacent molecules, which lowers the energy of the lowest excited state, leading to a red‐shifted emission.^[^ ^19^, ^20^ ^]^ Meanwhile, the applied force could also strengthen intermolecular interactions that effectively restrict intramolecular aromatic motions. By suppressing the non‐radiative pathways, compression can trigger a significant luminescence enhancement.^[^ ^21^, ^22^ ^]^ Notably, high pressure typically induces red‐shifted photoluminescence (PL) with reduced intensity, a phenomenon known as pressure‐induced emission quenching (PIQ).^[^ ^23^, ^24^ ^]^ By contrast, pressure‐triggered blue‐shifted emission is rare, likely due to the strong intermolecular interactions during compression.^[^ ^25^, ^26^, ^27^ ^]^ Designing piezochromic organic materials that exhibit anomalous emission enhancement and hypsochromic responses thus remains a long‐standing issue. Moreover, an unresolved question is whether piezofluorochromic shifts can be predicted solely from ambient molecular structures, a capability that would greatly advance rational design of pressure‐responsive luminophores.

In this work, two aramid isomers of PP (CCDC: 2213660) and OO (CCDC: 2213663) isomers (C_15_H_15_NO_3_) are selected as prototype systems for high‐pressure investigation. By introducing methoxy substituents at different positions on the benzanilide backbone, we modulate the noncovalent conformational lock (NCL) and thereby control molecular conformation and crystal packing at ambient conditions (1 atm).^[^ ^12^ ^]^ With increasing pressure, the dynamic interplay of molecular contraction, planarization, and distortion leads to markedly different PL responses between the two compounds. Meanwhile, the peak‐pressure dependent irreversible PL changes upon decompression (r1 atm) underscore the potential of these materials for practical applications in pressure‐responsive devices and sensing technologies. Additionally, intermolecular rotation is proposed to be an important indicator for predicting the luminescence shifts of aramids at high pressure. Based on these findings, a new design strategy is proposed, in which conjugation modulation enables predictable and tunable piezofluorochromic responses in aramids. This work not only opens new avenues for the design of solid‐state organic luminophores but also highlights the pivotal role of molecular structure in controlling piezofluorochromism.

## 2 Results and Discussion

**Figure 1:**
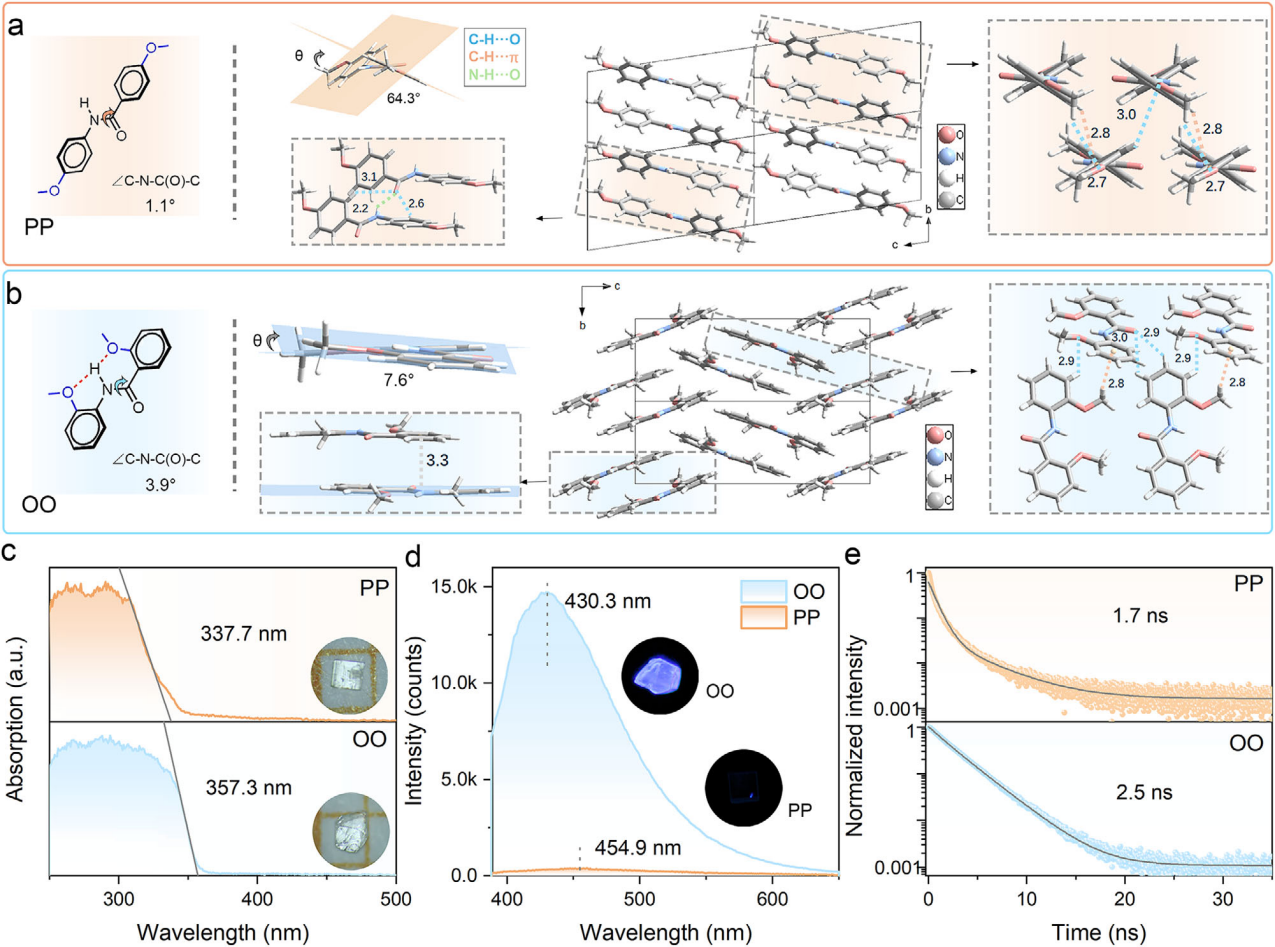
a,b) Ambient molecular conformations and crystal packing structures of a) PP and b) OO. The crystalline structures are derived from geometry optimizations of previously reported structures (CCDC: 2213660 and 2213663). c) UV–vis absorption spectra of PP (top) and OO (bottom) crystals at 1 atm. Insets are the photographs of as‐synthesized crystals under white‐light illumination. d) Ambient fluorescence spectra of PP and OO crystals under 365 nm excitation. Insets show the corresponding luminescence photographs. e) Luminescence decay curves of PP (top) and OO (bottom) crystals at 1 atm.

The aramid dyads PP and OO microcrystals used in this work are synthesized following previously reported procedures (see details in Schemes S1–S11, Supporting Information).^[^ ^12^ ^]^ At 1 atm, the position of methoxy substitution is deliberately varied via side‐chain engineering to modulate the NCL effect, thereby directing the resultant crystalline architectures. PP adopts a nonplanar molecular conformation, with a torsion angle of 1.1° around the C─N─C(O)─C bond and a dihedral angle of 64.3° between the two phenyl rings (**Figure** **1**a). The molecules assemble into layers through intermolecular hydrogen bonds (C─H∙∙∙O and N─H∙∙∙O) and C─H∙∙∙π interactions, involving terminal methoxy groups, central aramid fragments, and phenyl rings. These molecular sheets further stack along the *z*‐axis to form a 3D architecture. In contrast, OO exhibits strong intramolecular electrostatic interactions between central oxygen atoms and amide protons, forming five‐ and six‐membered hydrogen‐bonded rings within the molecule (Figure 1b). The side‐chain NCL effectively restricts intramolecular rotation, yielding a nearly planar backbone with a C─N─C(O)─C torsion angle of 3.9°, and a dihedral angle of only 7.6°. Regarding intermolecular organization, OO displays puckered molecular sheets within the *bc* plane, stabilized by a network of C─H∙∙∙O, N─H∙∙∙O, C─H∙∙∙π, and π–π stacking interactions. This extensive network of intra‐ and intermolecular interactions endows OO with a rigid and planar structure (Table S1, Supporting Information), in sharp contrast to the twisted and flexible conformation of PP. Previous theoretical studies confirm that the enhanced restriction of intramolecular rotation in OO, relative to PP, is evidenced by its significantly lower reorganization energy (RE) and root‐mean‐square deviation (RMSD).

These structural differences of PP and OO lead to their distinct optical properties at 1 atm. The two crystals display absorption edges at 337.7 nm and 357.3 nm, respectively, both yielding a colorless appearance (Figure 1c). The red‐shifted absorption in OO should arise from its more compact molecular packing with enhanced orbital coupling, as well as its more planar molecular conformation that facilitates intramolecular charge transfer (ICT).^[^ ^28^, ^29^ ^]^ Previous study has demonstrated that, compared to PP, the NCL‐induced planarity of OO not only strengthens ICT, but also suppresses intramolecular motion, resulting in significantly stronger luminescence for ambient OO (Figure 1d).^[^ ^12^ ^]^ The ICT‐based mechanism is further supported by the emission sensitivity to dielectric medium (Figure S1, Supporting Information). Notably, OO crystals display a prominent emission maximum at 430.3 nm, with a corresponding Commission Internationale de I'Eclairage (CIE) chromaticity coordinate of (0.174, 0.149) (Figure S2, Supporting Information). Compared to PP, the smaller Stokes shift of OO suggests reduced geometric distortion in excited state, consistent with its rigid structural nature (Table S2, Supporting Information). Furthermore, both compounds exhibit nanosecond‐scale luminescence lifetimes, indicative of their prompt fluorescence nature originating from singlet recombination (Figure 1e).

**Figure 2:**
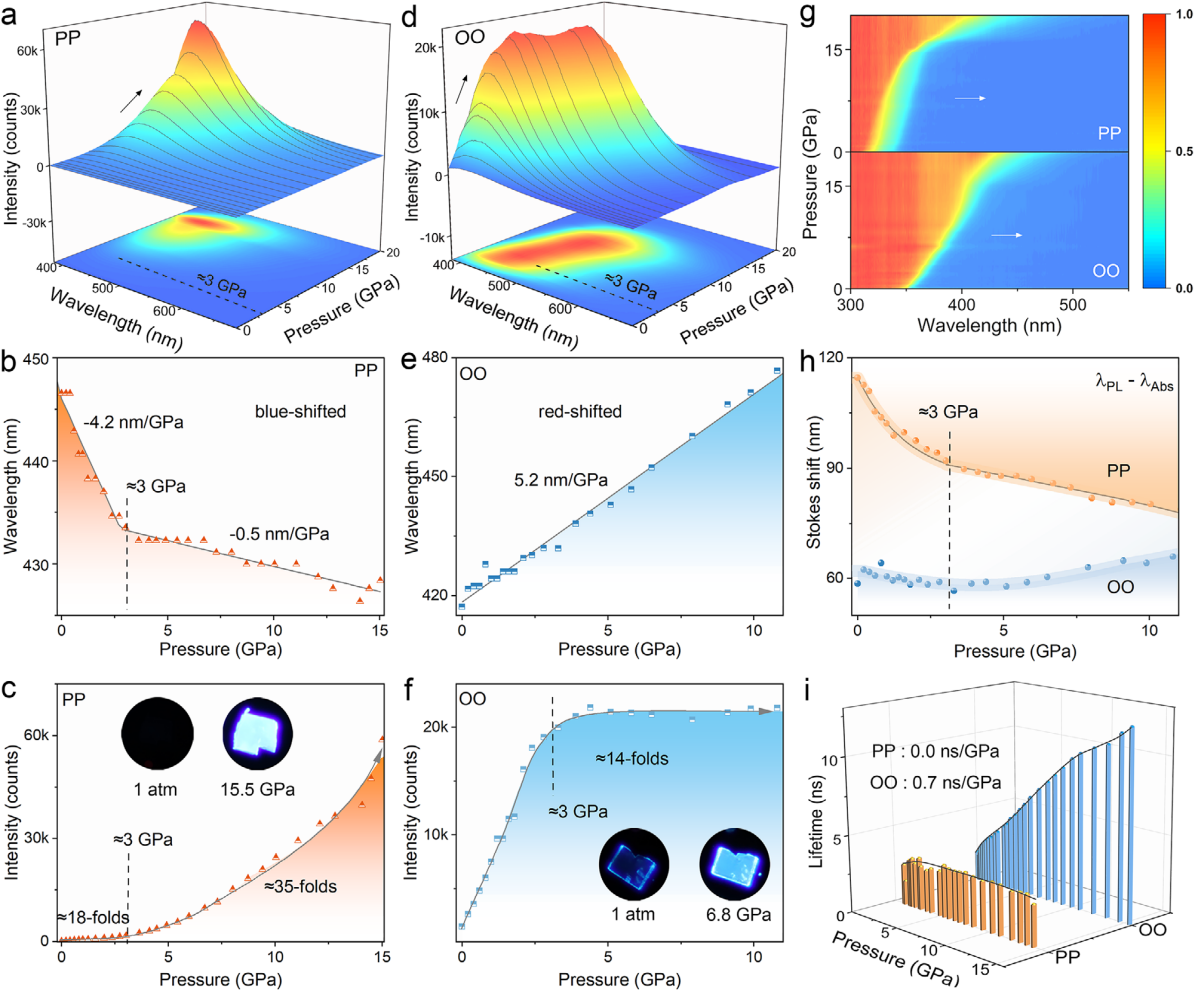
a) Luminescence evolution of PP crystal with increasing pressure. b,c) Pressure‐dependent variations in b) emission wavelength and c) emission intensity of PP crystal. Insets show the luminescence microphotographs of the detected crystal with identical exposure time. d) Luminescence spectra of OO crystal as a function of pressure. e,f) High‐pressure e) emission wavelength and f) emission intensity of OO crystal. Insets exhibit the luminescence microphotographs of the detected crystal. g) 2D projections of the absorption spectra of PP (top) and OO (bottom) crystals with pressure increases. h) Calculated Stokes shifts of PP and OO under compression. i) Evolution of luminescence lifetimes of PP and OO under high pressure.

High‐pressure luminescence measurements are first conducted on PP crystal, leveraging its flexible architecture that facilitates efficient modulation of photophysical properties (**Figure** **2**a–c). As pressure increases to ≈3 GPa, the fluorescence of PP exhibits an anomalous blueshift at −4.2 nm/GPa, accompanied by a moderate pressure‐induced emission enhancement (PIEE) of ≈18‐fold. Between ≈3 and ≈15 GPa, emission intensity rises sharply, alongside an additional ≈5 nm hypsochromic shift (−0.5 nm/GPa). Correspondingly, a vivid sky‐blue luminescence is observed at ≈15 GPa, characterized by a CIE chromaticity coordinate of (0.176, 0.150) (Figure S3, Supporting Information). The noticeably reduced pressure coefficient in this pressure range suggests a pressure‐driven structural contraction that increases structural rigidity. This enhanced rigidity likely suppresses molecular motions, thereby reducing nonradiative losses and contributing to the observed PIEE.^[^ ^30^, ^31^ ^]^ Meanwhile, the anomalous blueshift should result from a synergistic effect between strengthened intermolecular contacts and intramolecular deformation during compression.^[^ ^32^, ^33^, ^34^ ^]^ Moreover, the progressive narrowing of the full width at half maximum (FWHM) of the emission peak below ≈15 GPa points to a rare degeneracy of the emissive states in organic phosphors at high pressure (Figure S4, Supporting Information).^[^ ^35^ ^]^ This spectral narrowing may further support the observed hypsochromic shift with limited carrier transformation. Above ≈15 GPa, PP luminescence gradually diminishes and begins to exhibit a typical redshift (Figure 2a; Figure S5, Supporting Information). This behavior should be attributed to the excessive structural contraction and destruction, which enhances conjugation and orbital overlap, while simultaneously promoting the nonradiative energy dissipation. The accompanying spectral broadening in this pressure range further indicates the onset of structural amorphization (Figure S4, Supporting Information).

In contrast, OO exhibits sustained red‐shifted (≈11 nm) and intensified (≈14‐fold) luminescence under compression to ≈3 GPa, primarily due to substantial structural contraction (Figure 2d–f). Between ≈3 and ≈11 GPa, the emission redshifts by an additional ≈45 nm, gradually varying the emission color toward sky‐blue, while the emission intensity remains essentially constant. This stability suggests a dynamic equilibrium between PIEE and PIQ, associated with a complex interplay among structural contraction, molecular distortion, and inevitable structural destruction, as supported by the pronounced FWHM broadening (Figure S6, Supporting Information). Compared with PP, OO shows much larger emission shifts under high pressure, reflecting the influence of its more extensive interaction networks. Furthermore, the potential structural collapse above ≈11 GPa contributes to luminescence quenching and the continued redshifts (Figure S7, Supporting Information). At ≈17 GPa, only a considerably weak greenish emission remains in OO crystal (Figure S8, Supporting Information). The luminescence quenching is likely associated with enhanced nonradiative recombination under substantial compression, an effect that is further intensified by the deviatoric stress.

To evaluate the mechanism underlying the luminescence variations at high pressure, UV–vis absorption spectra and luminescence decay curves are recorded for both PP and OO crystals. As illustrated in Figure 2g and Figure S9 (Supporting Information), the absorption edges of both compounds keep sustaining redshifts upon compression. This behavior should be attributed to pressure‐induced structural contraction and conformational changes, which enhance orbital coupling and conjugation effects, as well as potentially promote the ICT process.^[^ ^28^, ^29^ ^]^ Importantly, the persistent redshift confirms that the blue‐shifted luminescence observed in PP is not associated with a twisted molecular conformation with reduced effective delocalization, a common cause of simultaneous hypsochromic shifts in both absorption and emission spectra.^[^ ^34^, ^36^ ^]^ To further explore excited‐state distortion under compression, high‐pressure Stokes shifts are calculated from the differences between emission and absorption wavelengths (Figure 2h). As pressure increases to ≈3 GPa, both PP and OO display gradual reductions in Stokes shift, indicating that structural relaxation in the excited state becomes increasingly restricted.^[^ ^37^ ^]^ This suppression is likely a consequence of strengthened intermolecular interactions and increased lattice rigidity under high pressure. Notably, as ≈3 GPa coincides with the solidification pressure of the pressure transmitting medium of silicon oil, the enhanced deviatoric stress may also promote the suppression of excited‐state distortion.^[^ ^38^, ^39^ ^]^ With further pressurization, the Stokes shift of PP continues to decline, consistent with the steadily increasing luminescence intensity. In contrast, OO shows a progressive increase in Stokes shift up to ≈11 GPa, aligning with its relatively constant emission intensity over this pressure range.

Regarding the pressure‐dependent evolution of luminescence decay curves (Figure 2i; Figures S10 and S11, Supporting Information), PP exhibits an essentially unchanged PL lifetime, despite a noticeable emission enhancement. According to the relation of *τ* = 1/(*k_nr_ + k_r_*), this behavior suggests a synergistic modulation between nonradiative (*k_nr_*) and radiative (*k_r_*) recombination rates. Pressure‐induced increases in molecular rigidity could restrict intramolecular motions, thereby suppressing nonradiative decay channels (decreased *k_nr_*).^[^ ^30^ ^]^ Simultaneously, potentially facilitated ICT or other electronic effects could also contribute to an increased radiative recombination (increased *k_r_*).^[^ ^12^ ^]^ In contrast, OO displays a marked elongation of luminescence lifetime in tandem with emission enhancement with increasing pressure. This trend suggests that fluctuations in nonradiative rate primarily govern the luminescence efficiency of high‐pressure OO.^[^ ^40^ ^]^ This phenomenon likely arises from a combination of suppressed intramolecular vibrations and pressure‐triggered disruption or rearrangement of intermolecular stacking.

**Figure 3:**
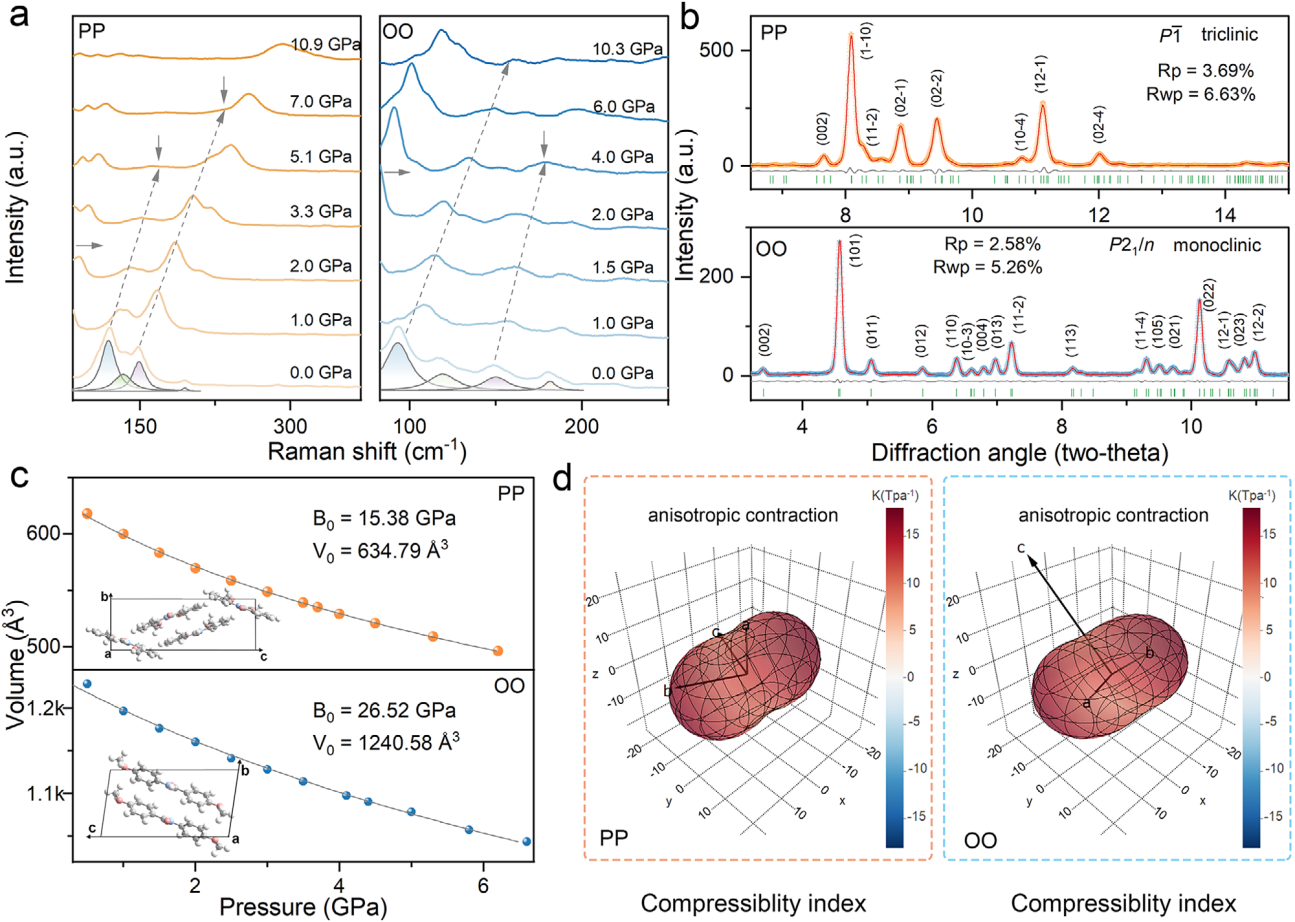
a) Pressure‐dependent evolution of low‐frequency Raman vibrations of both PP (left) and OO (right). b) Pawley refinement of the ambient ADXRD spectra of PP (up) and OO (down). The orange/blue and red lines represent detected and refined signal, respectively. The green bars and grey lines exhibit refined peak positions and the differences between detected and calculated results. c) Pressure‐dependent lattice volume (*P*–*V*) of PP (top) and OO (bottom). The solid lines exhibit the fitting results by the third‐order Birch‐Murnaghan equation of state. Insets show the corresponding unit cell structures. d) Compressibility index of PP (left) and OO (right) as the function of high pressure.

To elucidate the structural origins of pressure‐induced emission shifts and enhancements, a combination of high‐pressure Raman, Fourier transform‐infrared (FT‐IR), angle‐dispersive X‐ray diffraction (ADXRD) measurements, and theoretical calculations is employed, enabling insight into both local and long‐range structural evolution. As shown in **Figure** **3**a, the characteristic low‐frequency Raman modes of PP, primarily associated with skeletal vibrations involving intermolecular rotation and out‐of‐plane motions, exhibit pronounced mode freezing with increasing pressure (Figure 3a; Table S3 and Figure S12, Supporting Information). This suggests a progressive suppression of intramolecular motions, particularly out‐of‐plane phenyl rotations, under compression.^[^ ^41^ ^]^ In contrast, all the low‐frequency Raman modes of OO continuously weaken as pressure increases (Figure 3a; Table S4 and Figure S13, Supporting Information). These results reveal that the abnormal blueshift in PP fluorescence is closely linked to pressure‐induced restriction of intramolecular rotations with respect to the amide plane.^[^ ^42^, ^43^, ^44^ ^]^ Complementary FT‐IR measurements show persistent redshifts in N─H stretching vibrations for both PP and OO, signifying progressively strengthened hydrogen bonding interactions at elevated pressures (Figure S14, Supporting Information). The abnormal blueshifts under sufficient compression suggest the pressure‐induced conformation distortion. Meanwhile, the decreasing shift rate at higher pressures implies the onset of considerable structural rigidity upon compression (Figure S15, Supporting Information).

The ADXRD patterns (Figure 3b) confirm the ambient structures of PP as triclinic (space group *P*‐1) and OO as monoclinic (space group *P*2_1_/*n*), respectively. Upon increasing pressure, all the diffraction peaks shift toward higher angles, indicating continuous lattice contraction without detectable phase transitions in either compound (Figures S16 and S17, Supporting Information). The noticeable broadening and attenuation of diffraction peaks indicate pressure‐induced structural disorder, correlating with the fluorescence quenching observed under sufficient compression (Figure 2a,d). From pressure‐volume (*P*–*V*) relationships, the bulk moduli (*B*_0_) of PP and OO are determined to be 15.38 and 26.52 GPa, respectively (Figure 3c). The markedly higher *B*_0_ of OO reflects its intrinsically more rigid structure, arising from extensive intra‐ and intermolecular interactions. Meanwhile, both PP and OO display anisotropic contraction, with visibly greater compressibility along a direction close to the *b*‐axis (Figure 3d; Figures S18 and S19, Supporting Information). This phenomenon is attributable to the relatively loose molecular packing and weaker intermolecular interactions along the stacking direction (Figure S20, Supporting Information).

**Figure 4:**
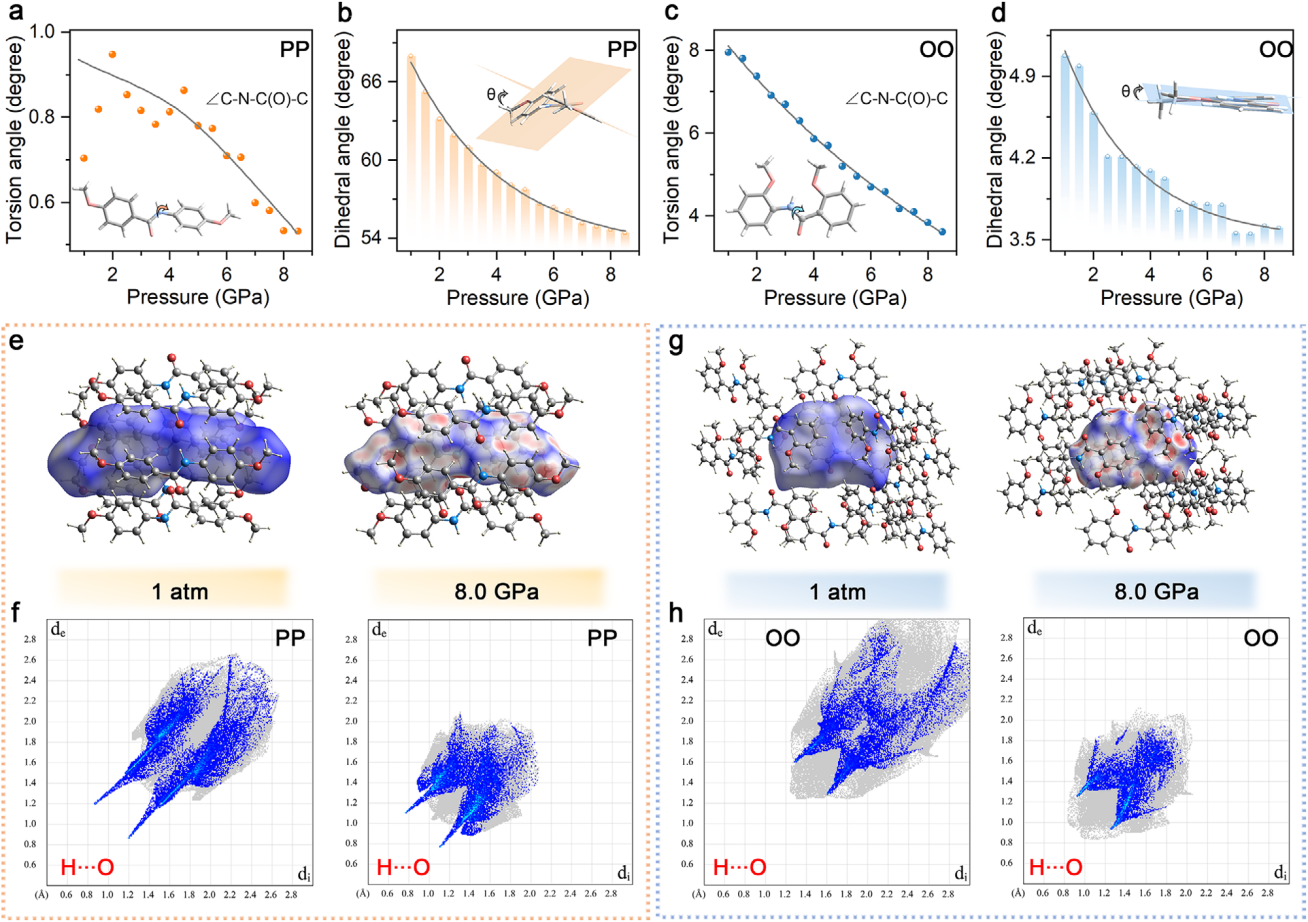
a,b) High‐pressure evolution of calculated a) intramolecular torsion angle around the C─N─C(O)─C linkage and b) dihedral angles between phenyl rings in PP. c,d) Pressure‐dependent c) intramolecular torsion angles around the C─N─C(O)─C linkage and d) dihedral angles between phenyl rings in OO. e) Hirshfeld surfaces of PP at 1 atm (left) and 8.0 GPa (right), respectively. f) 2D fingerprint plots of PP at 1 atm (left) and 8.0 GPa (right), showing the contributions from H∙∙∙O interactions. g) The Hirshfeld surfaces of OO at 1 atm (left) and 8.0 GPa (right), respectively. h) 2D fingerprint plots of OO at 1 atm (left) and 8.0 GPa (right) with the contribution of H∙∙∙O interactions only.

Building on the continuous structural contraction observed under high pressure, theoretical calculations are performed to resolve the detailed conformational changes. As illustrated in **Figure** **4**a–d, both PP and OO display progressively reduced torsion angles around the C─N─C(O)─C linkage, along with smaller dihedral angles between the two phenyl rings. These variations indicate a pressure‐induced planarization of molecular backbones in both systems. It is well‐known that increased planarity could enhance electron delocalization and promote intramolecular charge transfer, thereby improving orbital overlap and leading to redshifted absorption under compression (Figures S21 and S22, Supporting Information).^[^ ^28^, ^29^ ^]^ The anomalous blue‐shifted luminescence observed in PP, therefore, points to a competing interplay between this planarization effect and other structural factors. Meanwhile, pressure‐induced molecular contraction and planarization also decrease the excitation energy of the PP molecule, providing a direct contribution to the observed blue shift (Figure S23, Supporting Information).

Moreover, the markedly expanded red regions on the Hirshfeld surfaces of both PP and OO reveal substantially enhanced intermolecular interactions at high pressure (Figure 4e–h). In particular, the pronounced H∙∙∙O interactions, characterized by the spikes in the lower‐left region of 2D fingerprint plots, correspond to intermolecular hydrogen bonds.^[^ ^45^ ^]^ The pronounced reduction in (*d_i_* + *d_e_*) values further confirms the strengthening of hydrogen bonding networks, contributing to the increased structural rigidity at elevated pressures. This is consistent with the gradually decreased variation rates of fine structural parameters, as well as the visibly shortened H∙∙∙O and H∙∙∙π distances under pressure (Figures S24 and S25, Supporting Information). The resulting structural rigidification, coupled with strengthened intermolecular interactions, could effectively restrict intramolecular rotations, and limit geometric relaxation from excited to ground state. On one hand, this increased rigidity could facilitate exciton trapping in higher‐energy vibrational levels, resulting in the blue‐shifted luminescence in PP.^[^ ^41^, ^42^, ^43^ ^]^ On the other hand, the consequent suppression of nonradiative decay processes should account for the enhanced emission intensities of both PP and OO.^[^ ^30^, ^31^ ^]^ In the case of OO, the gradually reduced π–π overlap also contributes to the observed PIEE phenomenon (Figure S26, Supporting Information).^[^ ^46^ ^]^

**Figure 5:**
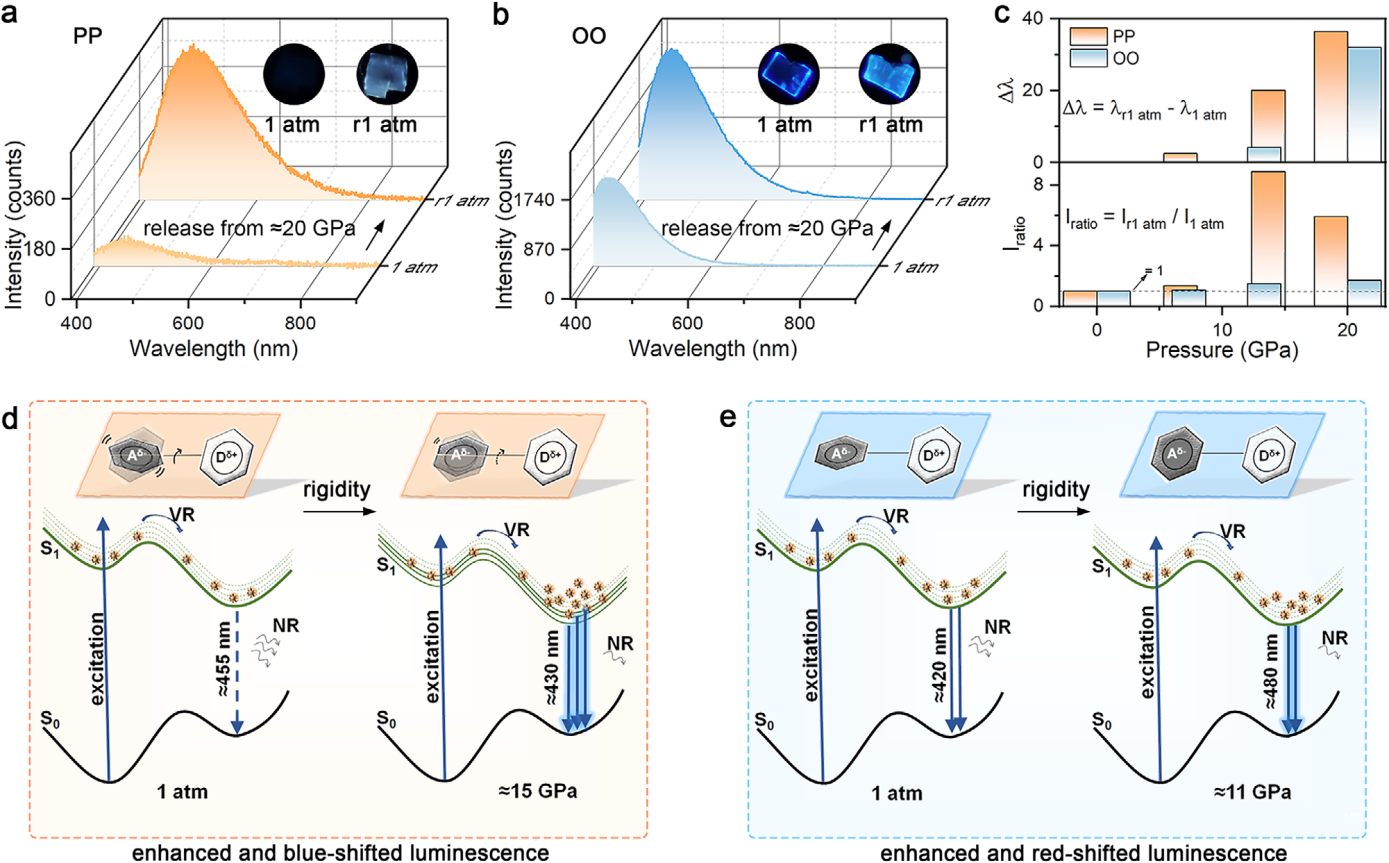
a,b) Comparisons between the luminescence spectra of (a) PP and (b) OO before and after high‐pressure treatment of ≈20 GPa. Insets reveal the luminescence microphotographs at 1 atm and r1 atm with identical exposure time. c) Evolution of the relative redshift in emission wavelength (top) and the intensity ratio (bottom) between the recovered and initial emission spectra. d) Proposed mechanism for pressure‐induced luminescence response of PP. e) Schematic illustration of the luminescence mechanism of OO across different pressure regimes.

Upon releasing pressure from ≈20 GPa, both PP and OO exhibit clearly red‐shifted and enhanced luminescence in their recovered crystals (**Figure** **5**a,b). High‐pressure treatment induces irreversible structural changes, resulting in intensified and visibly sky‐blue emission for both compounds. Correspondingly, the CIE chromaticity coordinates shift to (0.262, 0.326) for PP and (0.206, 0.231) for OO (Figure S27, Supporting Information). Meanwhile, the notable broadening and weakening of Raman, FT‐IR, and ADXRD peaks suggest that, while some local structural order is retained, long‐range crystallinity is significantly disrupted upon decompression (Figures S28 and S29, Supporting Information). The persistent shifts in N─H related vibrations further reveal the residual molecular distortion after pressure release. It is proposed that the applied external force disrupts the intermolecular stacking and interactions in quenched PP and OO, likely accompanied with residual molecular planarization at r1 atm.^[^ ^18^ ^]^ This structural reorganization could lower the energy of emissive states and convert previously forbidden transitions into an allowed singlet state, thereby enhancing emission efficiency and producing a redshift.^[^ ^47^ ^]^ Furthermore, since the extent of irreversible structural damage depends strongly on the maximum pressure applied, the recovered luminescence shows progressively larger redshifts and higher intensities as the peak pressure increases to ≈15 GPa (Figure 5c; Figures S30 and S31, Supporting Information). These findings suggest new avenues for exploiting PP and OO as pressure‐responsive smart materials for force‐sensing applications.

Based on combined experimental and theoretical analyses, the mechanisms underlying the pressure‐dependent fluorescence behavior of PP and OO, along with their structure‐property relationships, can be summarized as follows. As illustrated in Figure 5d, at 1 atm, PP molecules adopt a twisted, flexible conformation with randomly rotating phenyl rings. Upon photoexcitation, electrons in PP rapidly dissipate energy through internal conversion (IC) and vibrational relaxation (VR), eventually returning to the ground state (S_0_) from the lowest vibrational level of the excited singlet state (S_1_). Meanwhile, the highly dynamic structure of PP results in substantial nonradiative losses, producing considerably weak fluorescence centered ≈455 nm.^[^ ^12^ ^]^ As pressure increases, anisotropic structural contraction and denser molecular packing significantly strengthen intermolecular interactions, as evidenced by the pronounced red regions in the Hirshfeld surface maps. Concurrently, the PP backbone undergoes pressure‐induced planarization and rigidification, restricting intramolecular vibrations and phenyl rotations, as confirmed by the freezing of low‐frequency Raman modes (Figure 3a).^[^ ^30^, ^31^ ^]^ On the one hand, enhanced orbital coupling, stronger conjugative interactions, and a promoted ICT process could collectively narrow the optical bandgap with red‐shifted absorption edge.^[^ ^28^ ^]^ On the other hand, these structural responses could reduce geometric distortion in the excited state, as indicated by the decreased Stokes shifts (Figure 2h). Since Stokes shift reflects electron‐vibration coupling, and is proportional to the square of the displacement between potential energy surfaces in harmonic oscillator model. According to the energy gap law, this in turn, suppresses nonradiative decay pathways, thereby contributing to the PIEE observed in PP.^[^ ^48^ ^]^ Meanwhile, the calculated high‐pressure evolution of photoluminescence quantum yield (PLQY), alongside the *k_r_* and *k_nr_*, further supports this variation mechanism. The concurrent increase in *k_r_* and decrease in *k_nr_* collectively result in the increased emission efficiency observed at high pressure (Figure S32, Supporting Information). Additionally, the pronounced restriction of intramolecular motion could limit vibrational relaxation within the S_1_ state, promoting energy‐level degeneracy (as reflected by a decreased FWHM) and leaving a greater proportion of excited electrons in higher vibrational states.^[^ ^41^ ^]^ These electrons then recombine directly from higher vibrational levels to S_0_, resulting in a blue‐shifted fluorescence maximum at high pressure. In contrast, OO possesses an intrinsically planar and rigid molecular conformation stabilized by NCL (Figure 5e). This rigidity already enables efficient ICT and a certain degree of frontier‐orbital overlap, leading to relatively strong luminescence at 1 atm (Franck‐Condon principle). As a result, vibrational freezing under compression is minimal in OO. Instead, the applied pressure primarily induces molecular contraction with further planarization and rigidification, which strengthens intermolecular interactions and slightly reduces π‐π overlap between phenyl rings (as evidenced by the initially increased *k_r_* and decreased *k_nr_*). These changes lead to a typical bathochromic shift and intensified emission observed in high‐pressure OO. Upon sufficient compression, however, structural amorphization occurs. This process, accompanied by the extensive disruption of intermolecular interactions and the formation of structural defects, leads to a pronounced enhancement in *k_nr_* and the consequent quenching of luminescence.

**Figure 6:**
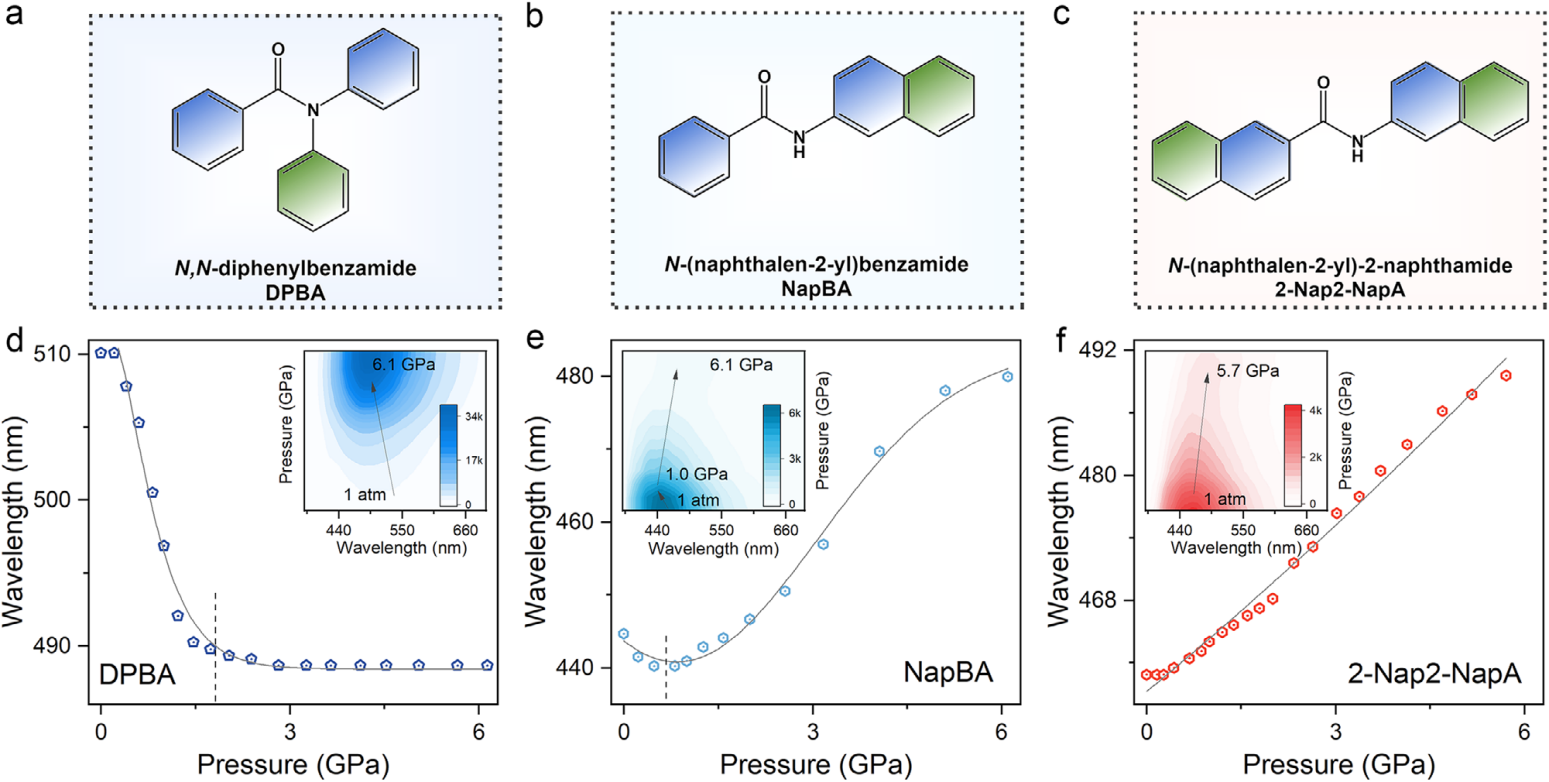
a–c) Molecular illustration of a) *N,N*‐diphenylbenzamide (DPBA), b) *N*‐(naphthalen‐2‐yl)benzamide (NapBA), and c) *N*‐(naphthalen‐2‐yl)‐2‐naphthamide (2‐Nap2‐NapA). d–f) Pressure‐dependent emission wavelength of d) DPBA, e) NapBA, and (f) 2‐Nap2‐NapA. Insets show the 2D projection of the emission spectra with increasing pressure.

As anticipated, intrinsic intramolecular free rotation emerges as a key structural factor responsible for the abnormal hypsochromic emission in aramid dyads upon compression. This understanding enables direct prediction of their piezofluorochromic behavior from the ambient molecular conformation alone, while also offering a strategy for deliberate luminescence tuning through molecular design. As a proof of concept, we propose a “conjugation modulation” approach to precisely regulate piezofluorochromism in these systems (**Figure** **6**a–f). The distinct piezofluorochromic responses arise from the combined influence of steric hindrance and molecular conjugation on intramolecular rotation. In the case of DPBA (*N,N*‐diphenylbenzamide), the phenyl substituent on the central aramid fragment introduces steric hindrance but fails to effectively restrict intramolecular rotation at 1 atm (Figure 6a). Consequently, DPBA exhibits a pronounced fluorescence blueshift under compression (Figure 6d). Its relatively low molecular symmetry, similar to that of PP, permits greater intramolecular rotational freedom, further contributing to the high‐pressure blue shift. By contrast, replacing one terminal phenyl ring with a naphthalene unit yields NapBA (*N*‐(naphthalen‐2‐yl)benzamide), where the moderately extended conjugation partially suppresses intramolecular motion (Figure 6b). NapBA displays only a slight blueshift under initial compression, likely arising from the counterbalance between enhanced rotational restriction and molecular contraction (Figure 6e). Finally, in 2‐Nap2‐NapA (*N*‐(naphthalen‐2‐yl)‐2‐naphthamide), substitution of both terminal phenyl rings with naphthalene groups markedly promotes π‐conjugation. The resulting extended molecular architecture is highly resistant to rotation, leading to continuously red‐shifted fluorescence upon compression (Figure 6c,f). Notably, the higher molecular symmetry of NapBA and 2‐Nap2‐NapA, similar to that of OO, may also contribute to their red‐shifted emission.

## 3 Conclusion

In summary, two aramid dyads, PP and OO, are investigated under high pressure, revealing distinct luminescence shifts with pronounced PIEE phenomena. In the absence of NCL, PP undergoes substantial increases in structural rigidity and planarity under compression, effectively freezing intramolecular rotations between the phenyl rings. The suppression of vibrational relaxation, combined with increased energy‐level degeneracy and a larger fraction of excited electrons remaining in higher vibrational states, results in an unusual blue‐shifted luminescence. In contrast, the NCL‐stabilized OO restricts intrinsic molecular vibration, leading to a conventional red‐shifted luminescence with increasing pressure. In both systems, the PIEE effect arises primarily from pressure‐triggered restrictions of molecular vibrations that suppress nonradiative decay pathways. Only for OO, additional luminescence enhancement is attributed to pressure‐driven changes in molecular stacking. Based on these insights, conjugation modulation is proposed as a design strategy to precisely control piezofluorochromism in aramid dyads. This work not only provides a foundational approach for predicting the high‐pressure behavior of aramid luminophores, but also offers a promising framework for developing AIE systems and advanced pressure‐responsive luminescent materials.

## Conflict of Interest

The authors declare no conflict of interest.

## Supporting information



Supporting Information

## Data Availability

The data that support the findings of this study are available from the corresponding author upon reasonable request.
