# Supplementary material for: Piezofluorochromism in Aramid Dyads: Pressure‐Triggered Luminescence Enhancement with Predictable Emission Shifts
Source: Adv Sci (Weinh). 2025 Nov 5;13(5):e18246. doi: 10.1002/advs.202518246 (PMC12850103; doi:10.1002/advs.202518246)
Supplement: Supplementary file 1 — Supporting Information [file ADVS-13-e18246-s001.docx]

Supporting Information

Piezofluorochromism in Aramid Dyads: Pressure-Triggered Luminescence Enhancement with Predictable Emission Shifts

Yayun Wang,^+[a]^ Zhe Jia,^+[a]^ Yaru Wang,^[a]^ Lin Wei,^[a]^ Zimin Hao,^[b]^ Yanan Wang,^[a]^ Jingwen Guo,^[a]^ Aisen Li,^[a]^ Lei Li,*^[b]^ Kai Wang,*^[a]^ and Qian Li*^[a]^

Dedication [+] These authors contributed equally to this work.

[a] Y. Wang, Z. Jia, Y. Wang, L. Wei, Y. Wang, J. Guo, Dr. A. Li, Prof. K. Wang, Prof. Q. Li
Key Laboratory of Quantum Materials Under Extreme Conditions in Shandong Province, School of Physics Science and Information Technology
Liaocheng University
Liaocheng, Shandong 252000, China
E-mail: kaiwang@lcu.edu.cn; liqian@lcu.edu.cn

[b] Z. Hao, Prof. L. Li
Shandong Key Laboratory of Applied Technology for Protein and Peptide Drugs, School of Pharmaceutical Sciences and Food Engineering
Liaocheng University
Liaocheng, Shandong 252000, China
E-mail: leili@lcu.edu.cn

***Material synthesis:***

All chemicals were obtained from commercial suppliers and used without further purification. The synthetic protocols of the five compounds of 4-methoxy-*N*-(4-methoxyphenyl)benzamide (**PP**), 2-methoxy-*N*-(2-methoxyphenyl)benzamide (**OO**), *N,N*-diphenylbenzamide (**DPBA**), *N*-(naphthalen-2-yl)benzamide (**NapBA**) and *N*-(naphthalen-2-yl)-2-naphthamide (**2-Nap-2-NapA**) have been reported in previous study, as illustrated in Scheme S1.^[1-4]^

**OO**

**Scheme S1.** The detailed synthesis routes of **PP**, **OO**, **DPBA**, **NapBA** and **2-Nap-2-NapA** at ambient conditions (DCM: dichloromethane, TEA: triethylamine).

Analytical nuclear magnetic resonance (NMR) spectra were applied to ensure the purification of the sample (Schemes S2 – S11). The spectra were recorded at 298 K, and on Bruker AVANCE III HD 500 and JEOL JNM-ECZ500R/S1 spectrometers.

**PP**^[1]^: ^1^H NMR (500 MHz, DMSO-*d*_6_) δ 9.98 (s, 1H), 7.95 (d, *J* = 9.0 Hz, 2H), 7.66 (d, *J* = 9.0 Hz, 2H), 7.05 (d, *J* = 9.0 Hz, 2H), 6.92 (d, *J* = 9.0 Hz, 2H), 3.83 (s, 3H), 3.74 (s, 3H). ^13^C NMR (125 MHz, DMSO-*d*_6_) δ 164.95, 162.21, 155.85, 132.86, 129.91, 127.53, 122.42, 114.14, 114.01, 55.86, 55.61.


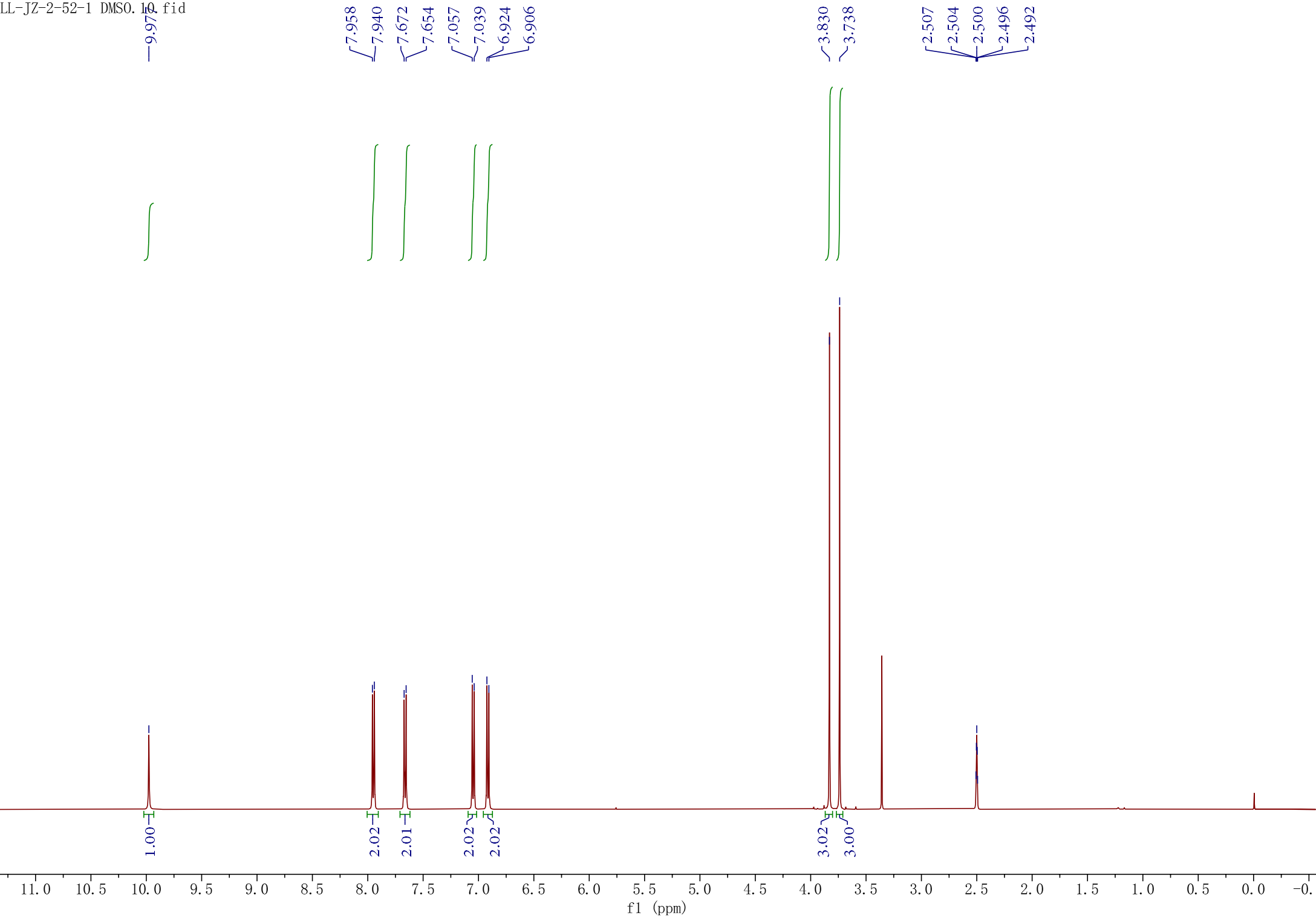


**Scheme S2.** The ^1^H NMR spectrum of **PP** (500 MHz, DMSO-*d6*).


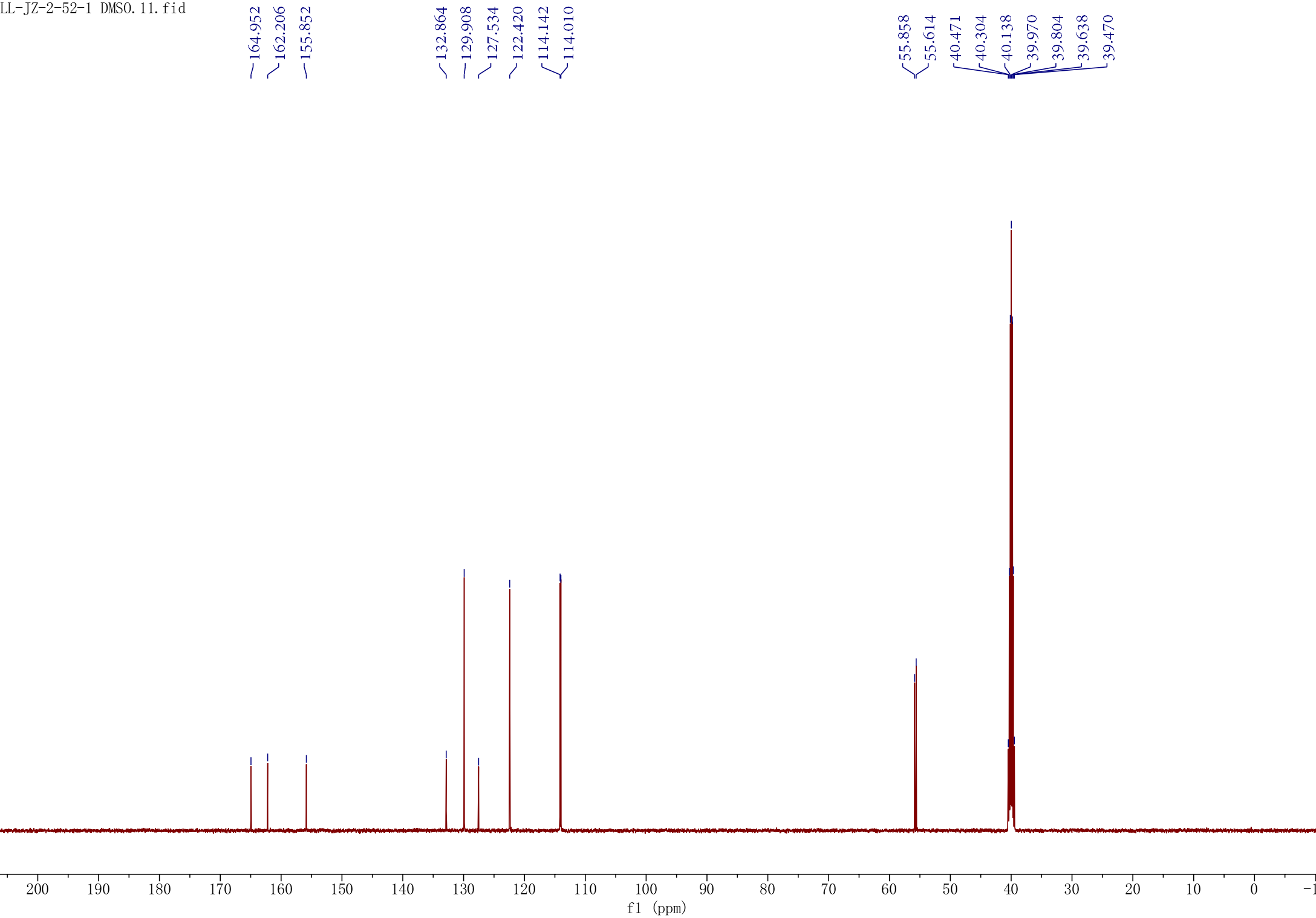


**Scheme S3.** The ^13^C NMR spectrum of **PP** (500 MHz, DMSO-*d6*).

**OO**^[1]^: ^1^H NMR (500 MHz, Chloroform-*d*) δ 10.59 (s, 1H), 8.65 (dd, *J* = 8.0, 2.0 Hz, 1H), 8.31 (dd, *J* = 8.0, 2.0 Hz, 1H), 7.50 – 7.45 (m, 1H), 7.15 – 7.10 (m, 1H), 7.08 – 6.99 (m, 3H), 6.91 (dd, *J* = 8.0, 2.0 Hz, 1H), 4.05 (s, 3H), 3.94 (s, 3H). ^13^C NMR (125 MHz, Chloroform-*d*) δ 162.91, 157.31, 148.30, 132.96, 132.35, 128.60, 123.36, 122.19, 121.42, 121.24, 120.27, 111.50, 109.96, 55.99, 55. 94.


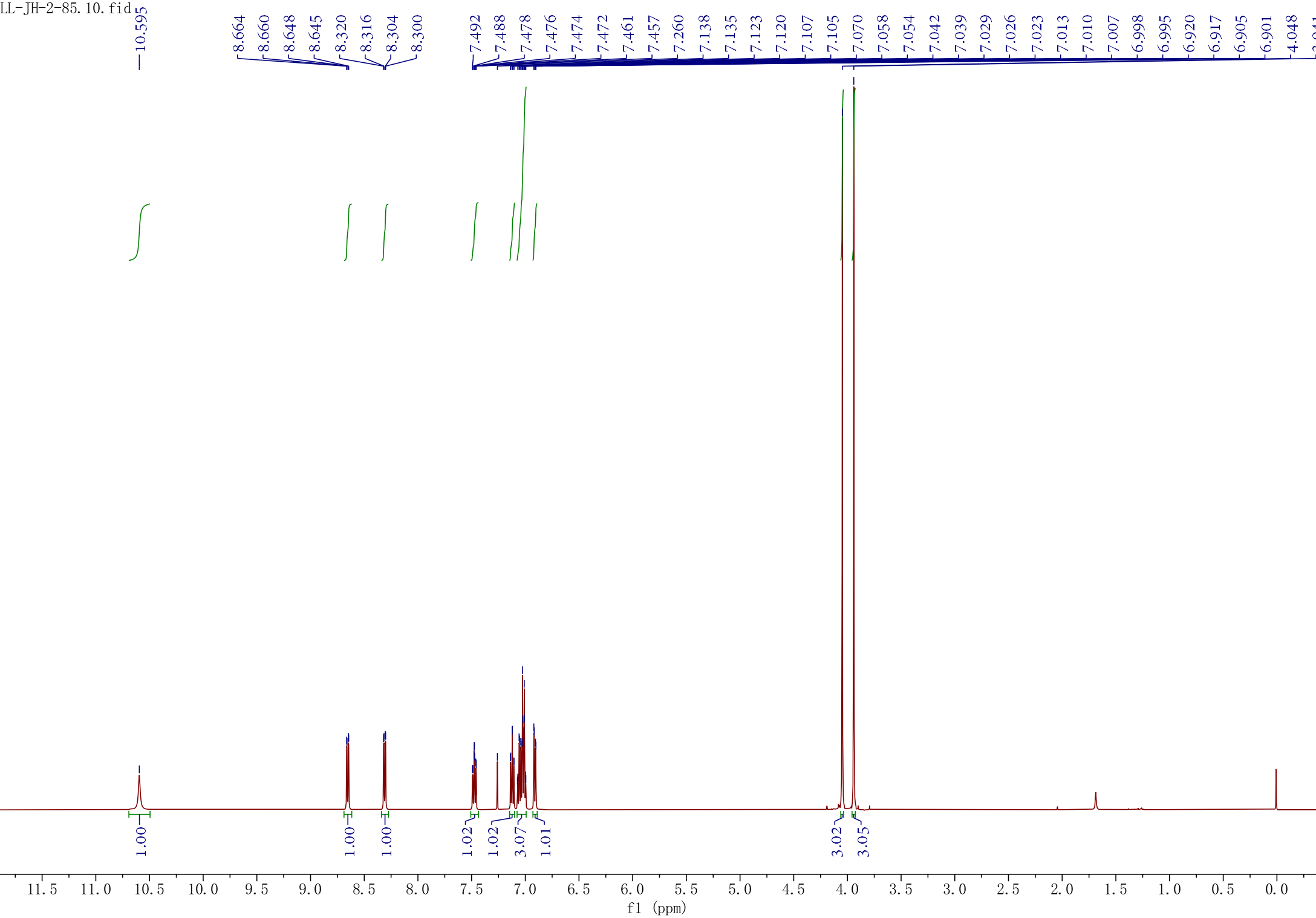


**Scheme S4.** The ^1^H NMR spectrum of **OO** (500 MHz, Chloroform-*d*).


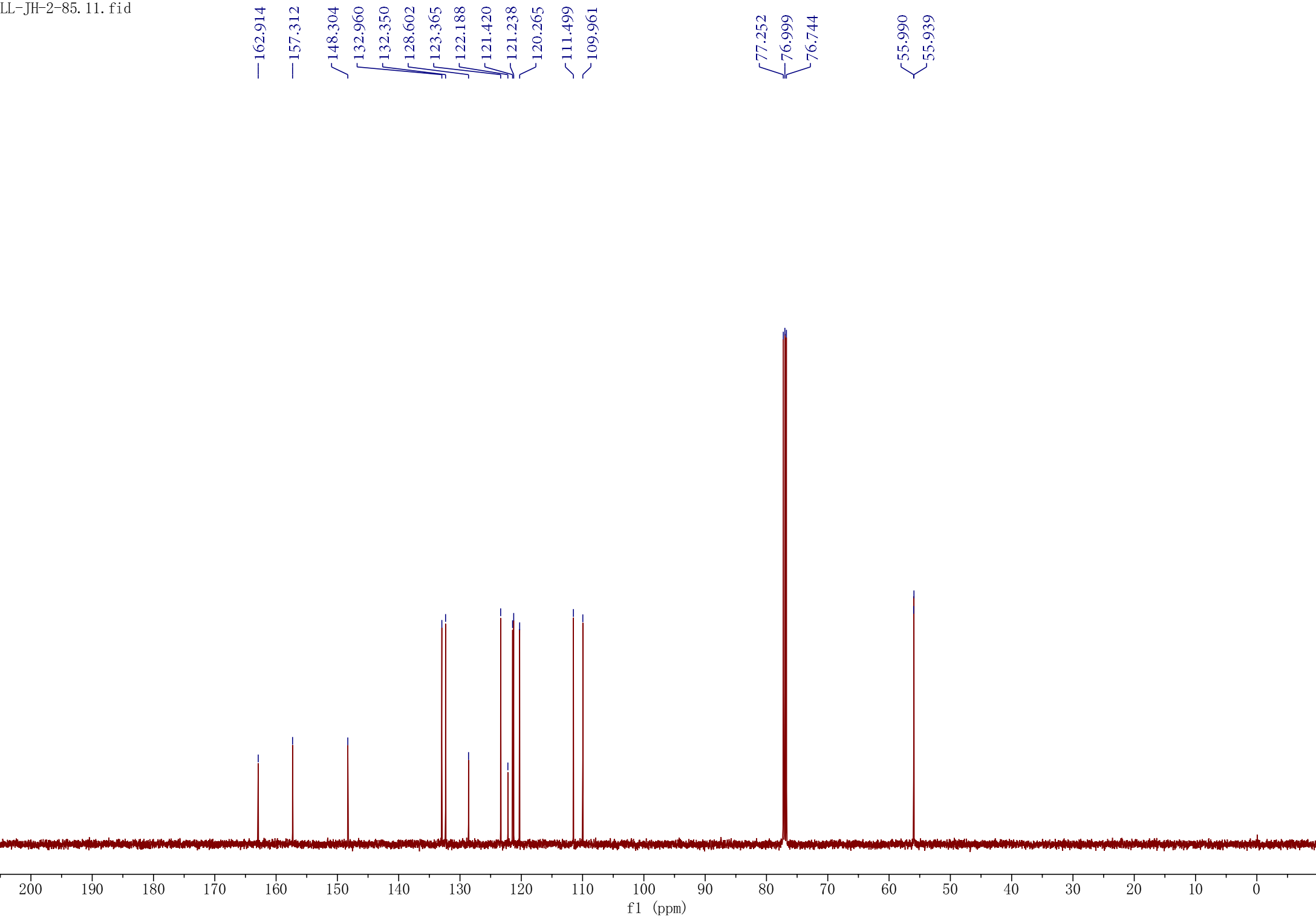


**Scheme S5.** The ^13^C NMR spectrum of **OO** (500 MHz, Chloroform-*d*).

**DPBA**^[2]^: ^1^H NMR (500 MHz, Chloroform-*d*) δ 7.48 – 7.44 (m, 2H), 7.32 – 7.26 (m, 5H), 7.24 – 7.13 (m, 8H). ^13^C NMR (125 MHz, Chloroform-*d*) δ 170.61, 143.88, 136.07, 130.13, 129.14, 129.07, 127.83, 127.46, 126.31.


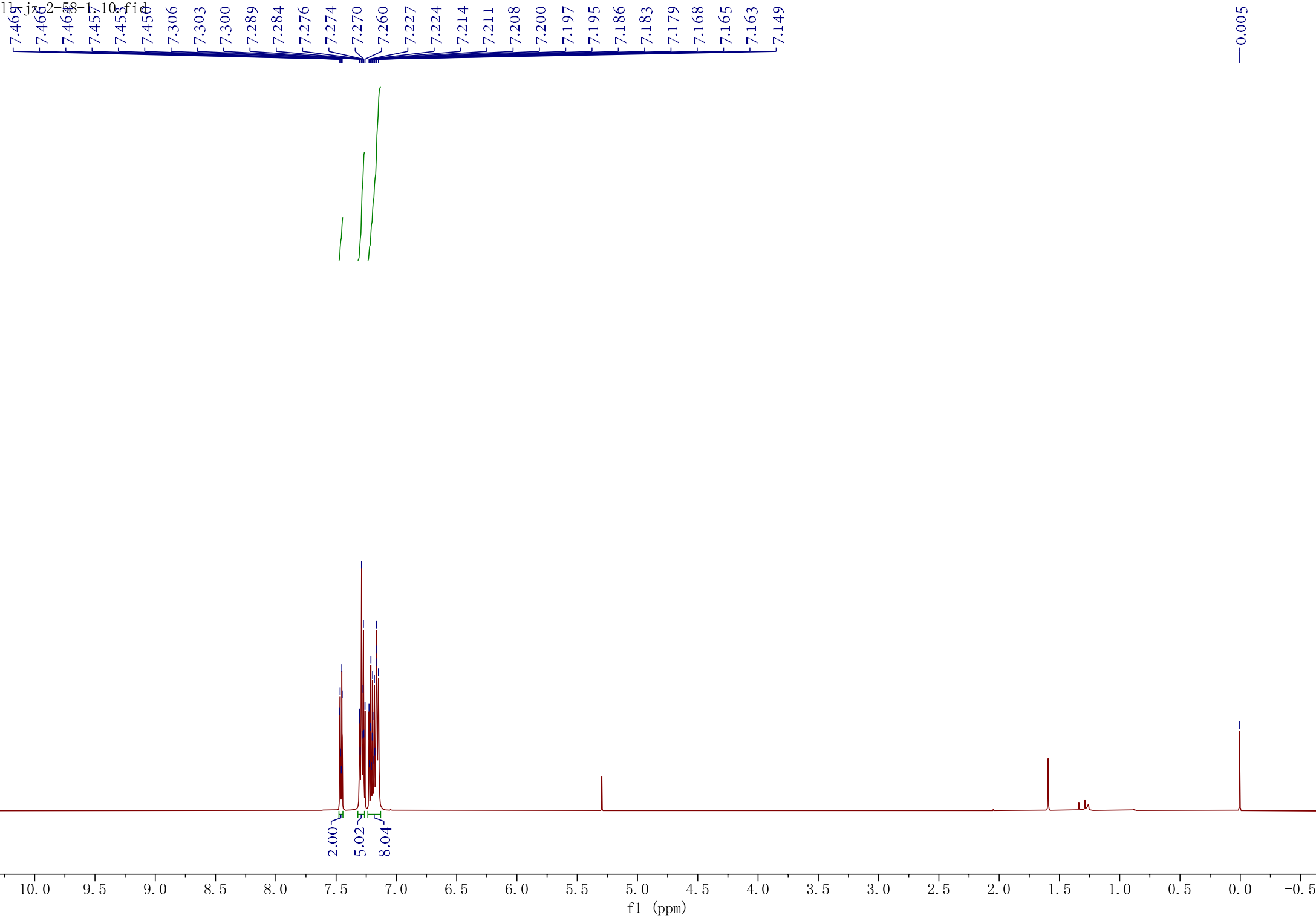


**Scheme S6.** The ^1^H NMR spectrum of **DPBA** (500 MHz, Chloroform-*d*).


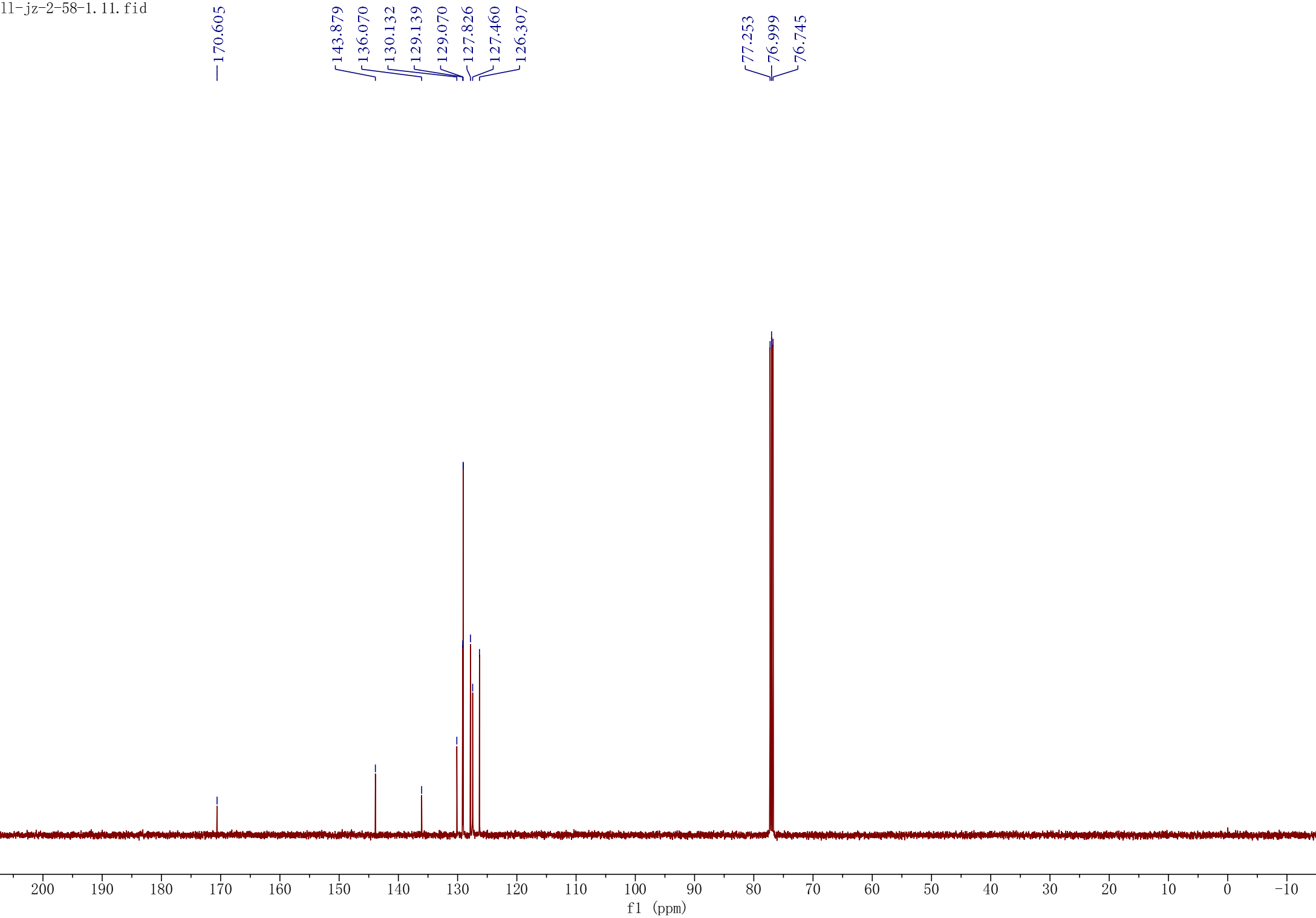


**Scheme S7.** The ^13^C NMR spectrum of **DPBA** (500 MHz, Chloroform-*d*).

**NapBA**^[3]^: ^1^H NMR (500 MHz, Chloroform-*d*) δ 8.33 (d, *J* = 2.0 Hz, 1H), 8.16 (s, 1H), 7.93 – 7.89 (m, 2H), 7.82 – 7.77 (m, 3H), 7.60 (dd, *J* = 8.5, 2.0 Hz, 1H), 7.57 – 7.52 (m, 1H), 7.49 – 7.40 (m, 4H). ^13^C NMR (125 MHz, Chloroform-*d*) δ 165.95, 135.37, 134.91, 133.82, 131.85, 130.76, 128.78, 128.76, 127.69, 127.54, 127.04, 126.50, 125.10, 120.15, 117.11.


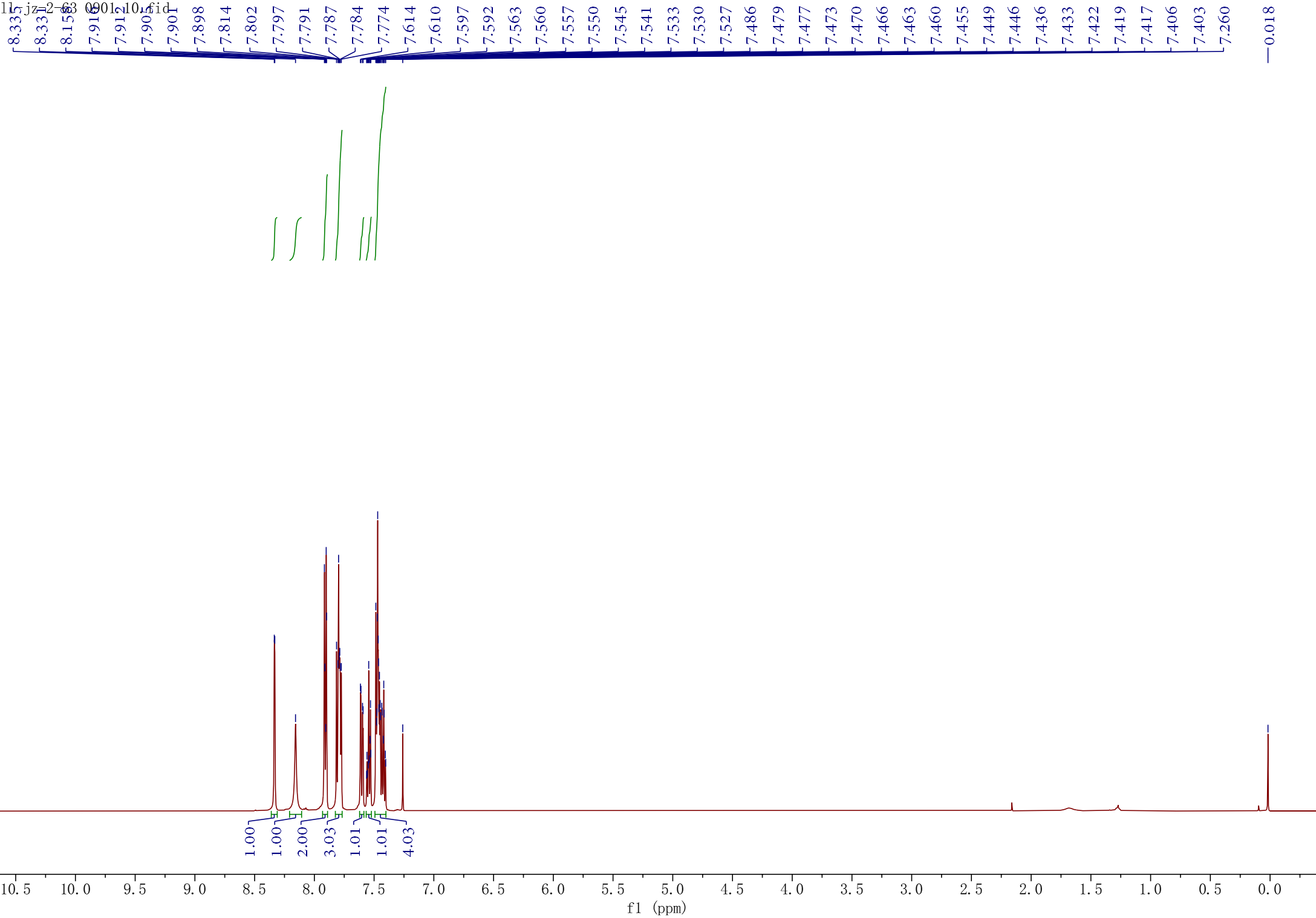


**Scheme S8.** The ^1^H NMR spectrum of **NapBA** (500 MHz, Chloroform-*d*).


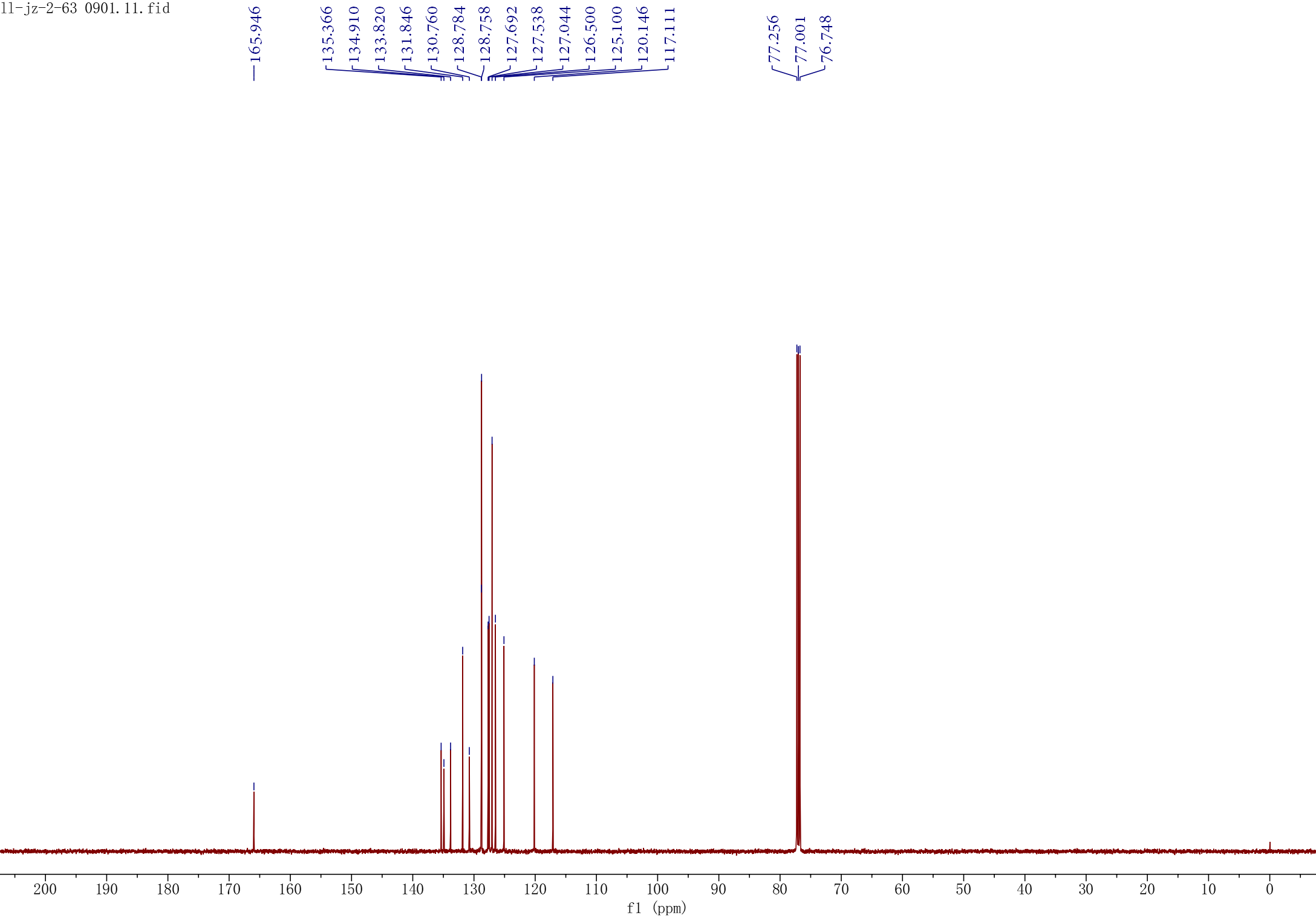


**Scheme S9.** The ^13^C NMR spectrum of **NapBA** (500 MHz, Chloroform-*d*).

**2-Nap-2-NapA**^[4]^: ^1^H NMR (500 MHz, DMSO-*d*_6_) δ 10.66 (s, 1H), 8.65 (sk, 1H), 8.52 (d, *J* = 2.0 Hz, 1H), 8.13 – 8.07kk (m, 1H), 8.09 (s, 2H), 8.05 – 8.01 (m, 1H), 7.94 (d, *J* = 9.0 Hz, 1H), 7.91 – 7.86 (m, 3H), 7.65 (tt, *J* = 6.9, 5.2 Hz, 2H), 7.53 – 7.48 (m, 1H), 7.46 – 7.42 (m, 1H). ^13^C NMR (125 MHz, DMSO-*d*_6_) δ 166.31, 137.36, 134.79, 133.84, 132.72, 132.57, 130.49, 129.46, 128.69, 128.55, 128.52, 128.36, 128.18, 127.96, 127.91, 127.38, 126.90, 125.28, 124.98, 121.43, 117.03.


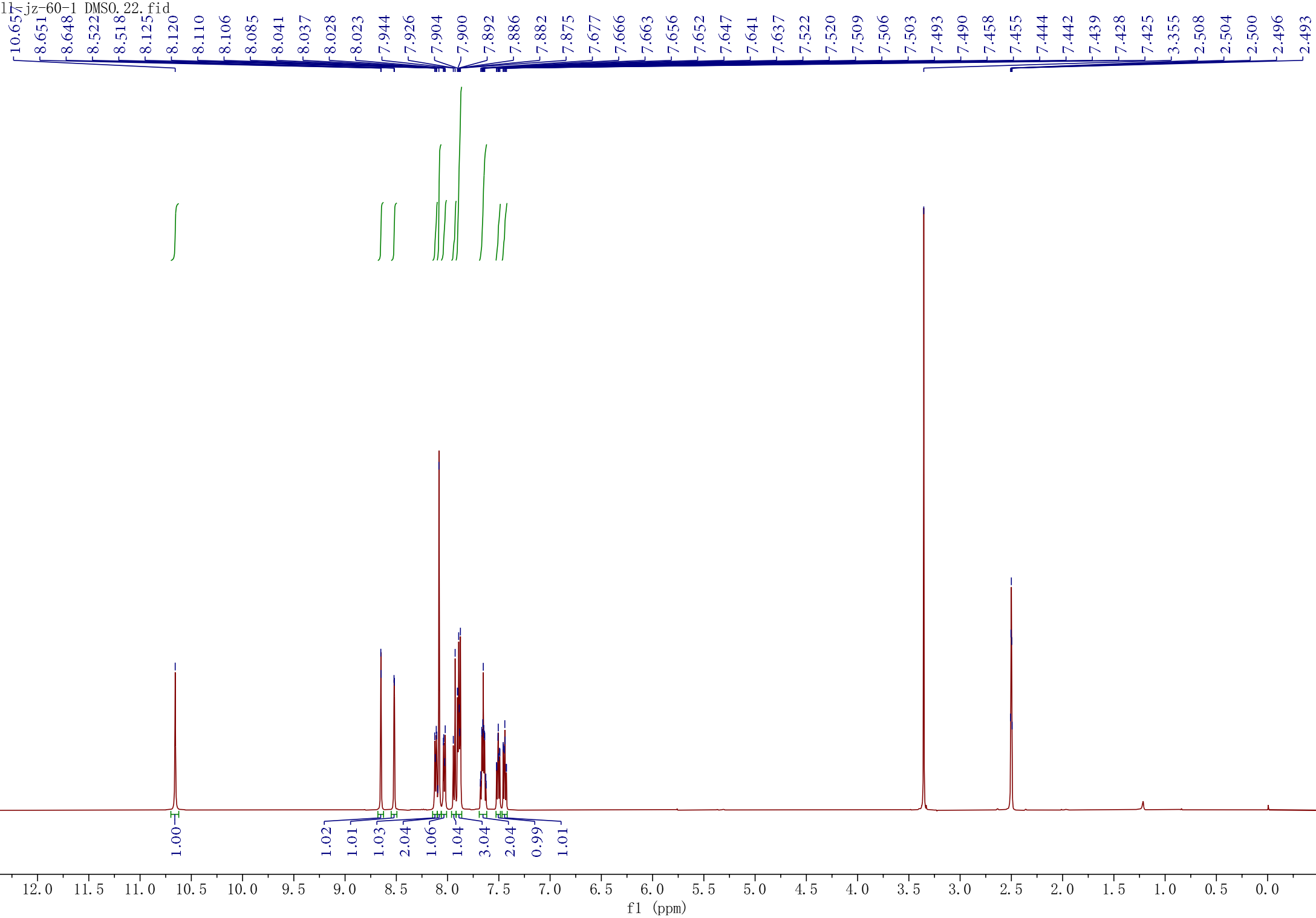


**Scheme S10.** The ^1^H NMR spectrum of **2-Nap-2-NapA** (500 MHz, DMSO-*d6*).


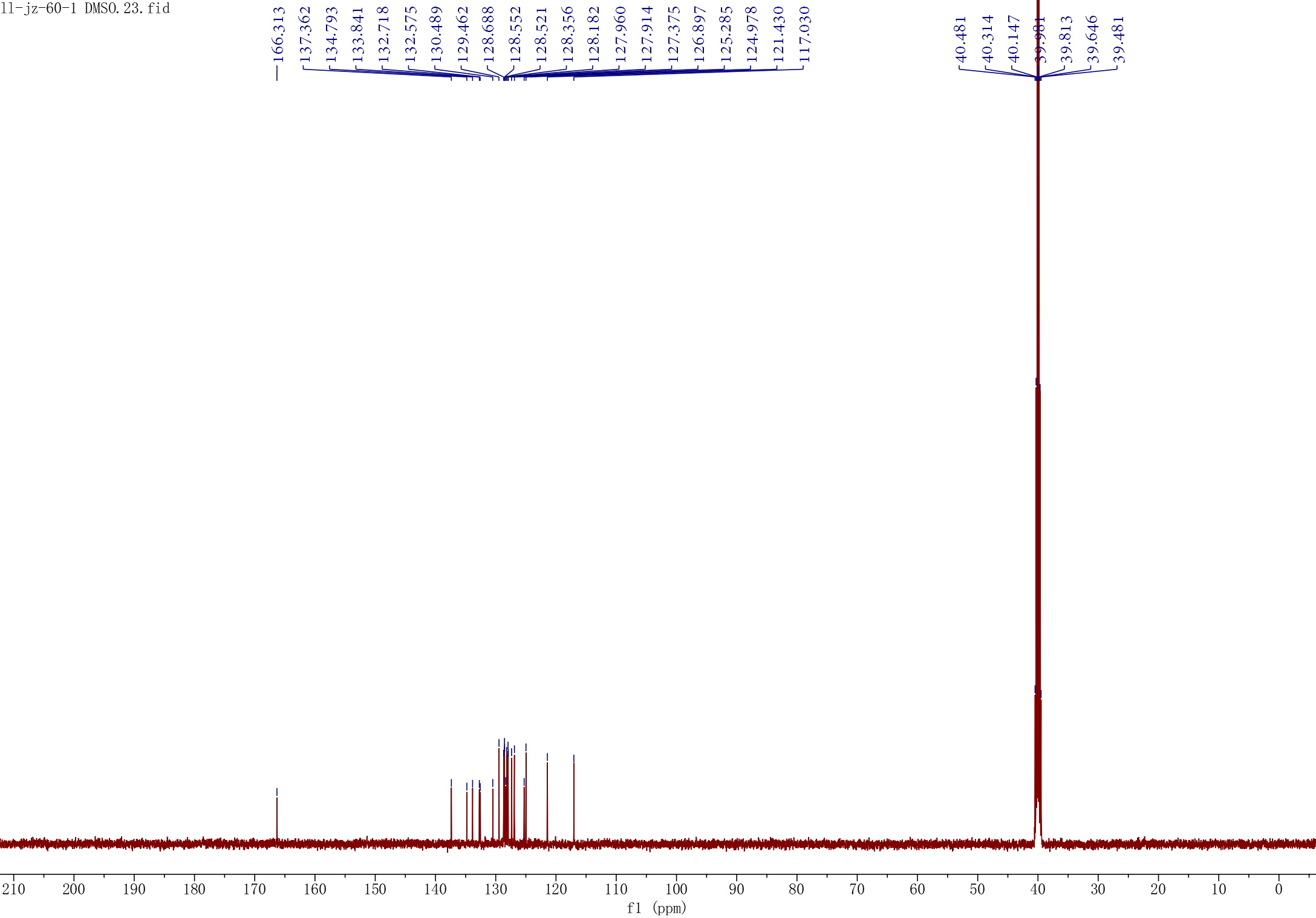


**Scheme S11.** The ^13^C NMR spectrum of **2-Nap-2-NapA** (500 MHz, DMSO-*d6*).

***Experiments***

*High-pressure sample loading*

High-pressure environment in this work was generated using a piston-cylinder symmetric diamond anvil cell (DAC) equipped with a pair of 400-micrometer-diameter anvils. A cylindrical hole (150 μm in diameter) was drilled at the center of the pre-indented T301 stainless steel gasket, serving as sample chamber. Pressure calibration was performed using ruby fluorescence method.^[5]^ Silicone oil (Aldrich, viscosity ~150 mPa·s) was used as the pressure-transmitting medium, with KBr employed exclusively for Fourier transform-infrared (FT-IR) spectra detections.^[6]^ All measurements were carried out at room temperature.

*High-pressure optical detections*

Photoluminescence (PL) emission spectra were acquired under 365 nm excitation using a high-performance Ocean Insight spectrometer. The *in-situ* microscopic optical path was provided by Liaocheng Light & Microvision Industrial Technology Co., Ltd. Corresponding PL micrographs were recorded with a Canon digital single lens reflex integrated with the micro-light-path, under consistent exposure conditions. Time-resolved luminescence decay curves were achieved under 375 nm excitation, using a PicoQuant PMA Hybrid single-photon counting module coupled to a Zolix Omni-λ3027i spectrometer. UV-Vis absorption spectroscopy under high pressure was conducted using an Ocean Insight QE65 Pro spectrometer with a DH-2000-BAL deuterium-halogen light source. Fourier transform-infrared (FT-IR) measurements were performed using a SHIMADZU IRTracer-100 microscope spectrometer equipped with a liquid-nitrogen-cooled detector. Raman experiments were carried out under the laser excitation at 785 nm, using HORIBA iHR550 spectrometer with a high sensitivity thermoelectrically cooled CCD detector. Angle-dispersive X-ray diffraction (ADXRD) experiments were performed at the beamline 15U1 of the Shanghai Synchrotron Radiation Facility (SSRF) using a monochromatic X-ray wavelength of 0.6199 Å. The instrument geometry was calibrated using a CeO₂ standard.

***Data analyses***

The high-pressure Stokes shifts were estimated based on the wavelengths of absorption edge and the center of emission peak.

The PL decay curves were fitted using double exponential function of

$$I\left( t \right)=I\left( 0 \right)\cdot\left[ A_{1}\cdot exp(-t/\tau_{1})+A_{2}\cdot exp(-t/\tau_{2}) \right]$$

in which *I* represented the PL intensity, *t* was the time after excitation, *τ*_1_ and *τ*_2_ showed the lifetimes for exponential components, respectively. The parameters of *I*(0), *A*_1_ and *A*_2_ were all constants for fitting. Then, the average lifetimes were obtained by the function of

$$\left\langle\tau\right\rangle=\left[ A_{1}{\tau_{1}}^{2}+A_{2}{\tau_{2}}^{2} \right]/\left[ A_{1}\tau_{1}+A_{2}\tau_{2} \right]$$

The two-dimensional diffraction patterns were processed into one-dimensional spectra using DIOPTAS software.^[7]^ The high-pressure lattice parameters were determined via Pawley refinements of ADXRD patterns with the software of Materials Studio. The pressure-volume (*P*-*V*) data were fitted by the third-order Birch-Murnaghan equation of state of

$$P(V)=\frac{3B_{0}}{2}\left[ \left( \frac{V_{0}}{V} \right)^{\frac{7}{3}}-\left( \frac{V_{0}}{V} \right)^{\frac{5}{3}} \right]\times\left\{ 1+\frac{3}{4}\left( B_{0}^{,}-4 \right)\left[ \left( \frac{V_{0}}{V} \right)^{\frac{2}{3}}-1 \right] \right\}$$

where *V*_0_, *B*_0_ and *B*_0_’ represented ambient lattice volume, bulk modulus and pressure derivative, respectively.

The axes compressibility of *K_l_* was calculated using the Principal Axis Strain Calculator software package, according to the following equation as:

$$K_{l}=-\frac{1}{l}\left( \frac{\partial l}{\partial P} \right)_{T}$$

where *l* was the lattice parameter along a given crystallographic axis, and *P* was the applied pressure. Note that the principal axes were determined as the eigenvectors of the full strain tensor.^[8]^

The energy gap law reveals the relationship between nonradiative decay rate constant of *k_nr_* from the excited state (*T*) to the ground state (*S*) as follows:^[8]^

$$k_{nr}\propto e^{-\beta[E\left( T_{m} \right)-E(S_{0})]}$$

The calculation of pressure-dependent evolution of photoluminescence quantum yield (PLQY), radiative rate (*k_r_*) and nonradiative rate (*k_nr_*) was shown as below:^[9]^

$$\Phi=\Phi_{0}\frac{\int F(\lambda_{em})}{\int F_{0}\lambda_{em}}\frac{A_{0}(\lambda_{ex})}{A(\lambda_{ex})}\frac{n^{2}}{{n_{0}}^{2}}$$

where *Φ* was the final PLQY, $\int F(\lambda_{em})$ represented the integration of photoluminescence (PL) intensity within the same interval. $A(\lambda_{ex})$ was the absorbance of the sample at the experimental excitation wavelength, and *n* was the refractive index. The subscript 0 of the parameter represented initial figure under ambient conditions. The ambient $\Phi_{0}$ values of PP and OO were obtained from previous studies, fixed at 0.07% and 66.8%, respectively.^[1]^

Meanwhile, the ratio of $\frac{n^{2}}{{n_{0}}^{2}}$ served as correction factor which was derived from the point source approximation, and parameter *n* could be calculated using the Clausius-Mossotti equation and Lorentz-Lorenz equation:

$$\frac{n^{2}-1}{n^{2}+2}\cdot\frac{1}{\rho}=\frac{4\pi}{3}\cdot N_{A}\cdot\alpha=R_{LL}$$

where $R_{LL}$ was the Lorentz-Lorenz constant which was determined by polarizability $\alpha$. And density $\rho$ could be calculated by cell volume according to the XRD refinement. After acquiring the corresponding PLQY and $\tau$, the radiative rates and nonradiative rates at high pressure were calculated from the following equations:

$$PLQY=\frac{k_{r}}{k_{r}+k_{nr}}$$

$$\tau=\frac{1}{k_{r}+k_{nr}}$$

***Theoritical calculation***

The fine structural information at high pressure was calculated using the CASTEP code within Materials Studio program, based on the pseudopotential plane-wave method and density functional theory. The geometry optimization was performed with local density approximation exchange-functional, using the convergence tolerance 5 × 10^-6^ eV/atom energy, 0.01 eV/Å max force, 0.02 GPa max stress, 5 × 10^-4^ Å max displacement, 100 maximum iterations and LBFGS algorithm. Norm-conserving pseudopotential was applied during calculation. The pressure applied was the equivalent hydrostatic pressure. The Raman and IR vibrations at ambient conditions (1 atm), as well as the frontier molecular orbitals and excitation energy at high pressure were calculated using the Gaussian program based on the optimized molecular structure at B3LYP/6-31G(d) level. The calculation of excitation energy was performed based on the single molecule only, and without the geometry optimization of excited state. Hirshfeld surface was calculated using the software CrystalExplorer.

| **Sample** | **Interactions** | **Interactions** | **Distance (Å)** |
| --- | --- | --- | --- |
| **PP** | Intermolecular  hydrogen bonding | C_5_-H∙∙∙O_3_ | 2.7 |
|  |  | C_6_-H∙∙∙O_2_ | 3.1 |
|  |  | C_9_-H∙∙∙O_2_ | 2.6 |
|  |  | C_12_-H∙∙∙O_1_ | 3.0 |
|  |  | N_1_-H∙∙∙O_2_ | 2.2 |
|  | C-H∙∙∙π | C_14_-H∙∙∙π | 2.8 |
| **OO** | Intermolecular  hydrogen bonding | C_10_-H∙∙∙O_2_ | 3.0 |
|  |  | C_11_-H∙∙∙O_2_ | 2.9 |
|  |  | C_12_-H∙∙∙O_3_ | 2.9 |
|  | Intramolecular  hydrogen bonding | N_1_-H∙∙∙O_1_ | 1.9 |
|  |  | N_1_-H∙∙∙O_3_ | 2.2 |
|  | C-H∙∙∙π | C_14_-H∙∙∙π | 2.8 |
|  | π–π | π–π | 3.7 |

**Table S1.** Comparisons between the dihedral angles and intermolecular distances in PP and OO at ambient conditions.

**
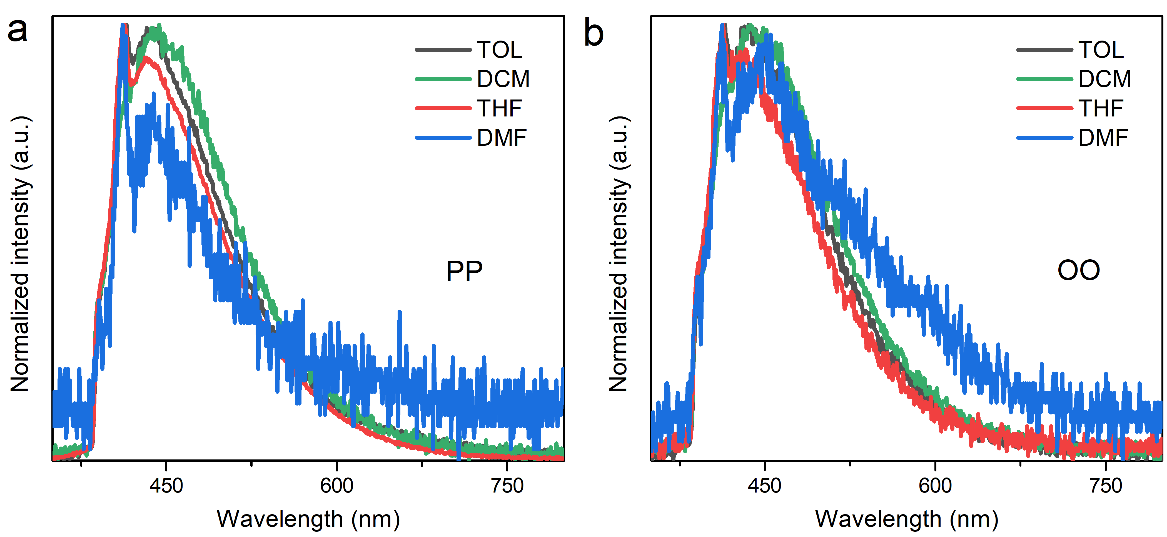
**

**Figure S1.** The luminescence spectra of (a) PP and (b) OO in dilute solutions with the similar concentration (*λ*_ex_ = 365 nm, *c* = 10^-4^ M). The abbreviation of TOL, DCM, THF and DMF are used to illustrate toluene, dichloromethane, tetrahydrofuran and dimethylformamide, respectively.

**
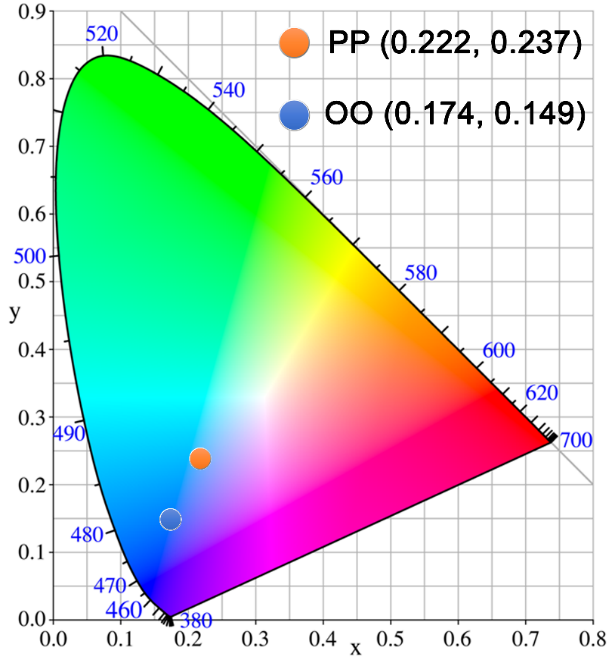
**

**Figure S2.** The Commission Internationale de I'Eclairage (CIE) chromaticity coordinates of PP and OO crystals at ambient conditions.

| **Compound** | **Absorption edge (nm)** | **Emission center (nm)** | **Stokes shift (nm)** |
| --- | --- | --- | --- |
| **PP** | 337.7 | 454.9 | 117.2 |
| **OO** | 357.3 | 430.3 | 73.0 |

**Table S2.** The absorption and emission wavelengths, as well as the calculated Stokes shifts of PP and OO. The Stokes shifts in this work are calculated based on the difference between absorption (bandgap) and emission wavelengths.

**
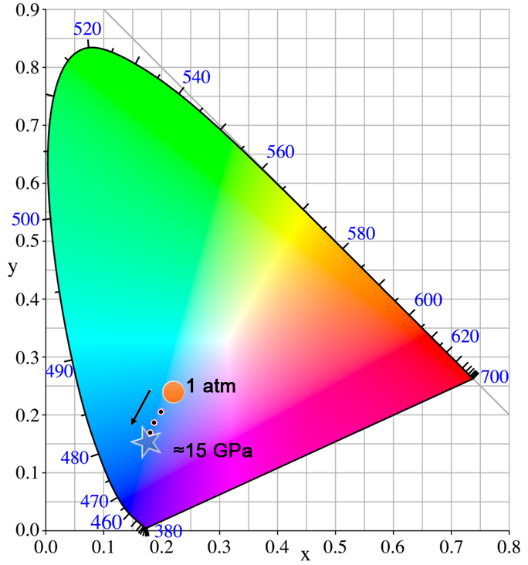
**

**Figure S3.** The evolution of typical CIE chromaticity coordinate of PP from 1 atm to ≈15 GPa.

**
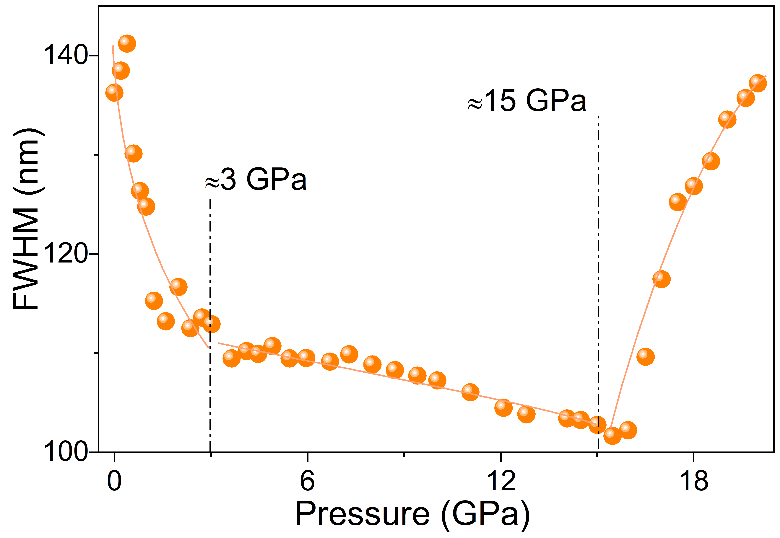
**

**Figure S4.** Pressure-dependent full width at half maximum (FWHM) of the emission spectra of PP.

**
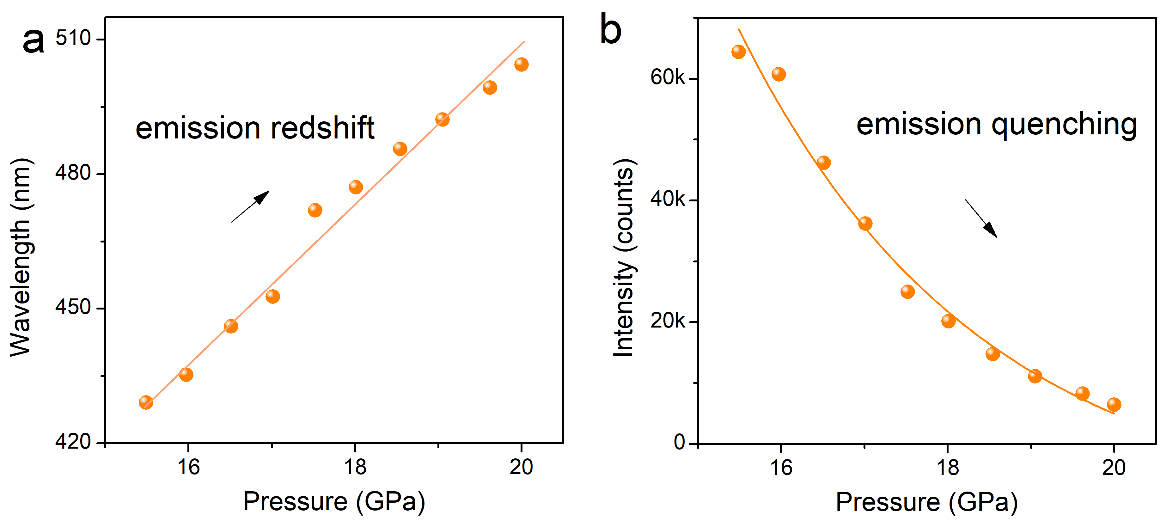
**

**Figure S5.** High-pressure evolution of emission (a) wavelength and (b) intensity of PP between ≈15 GPa and ≈20 GPa.

**
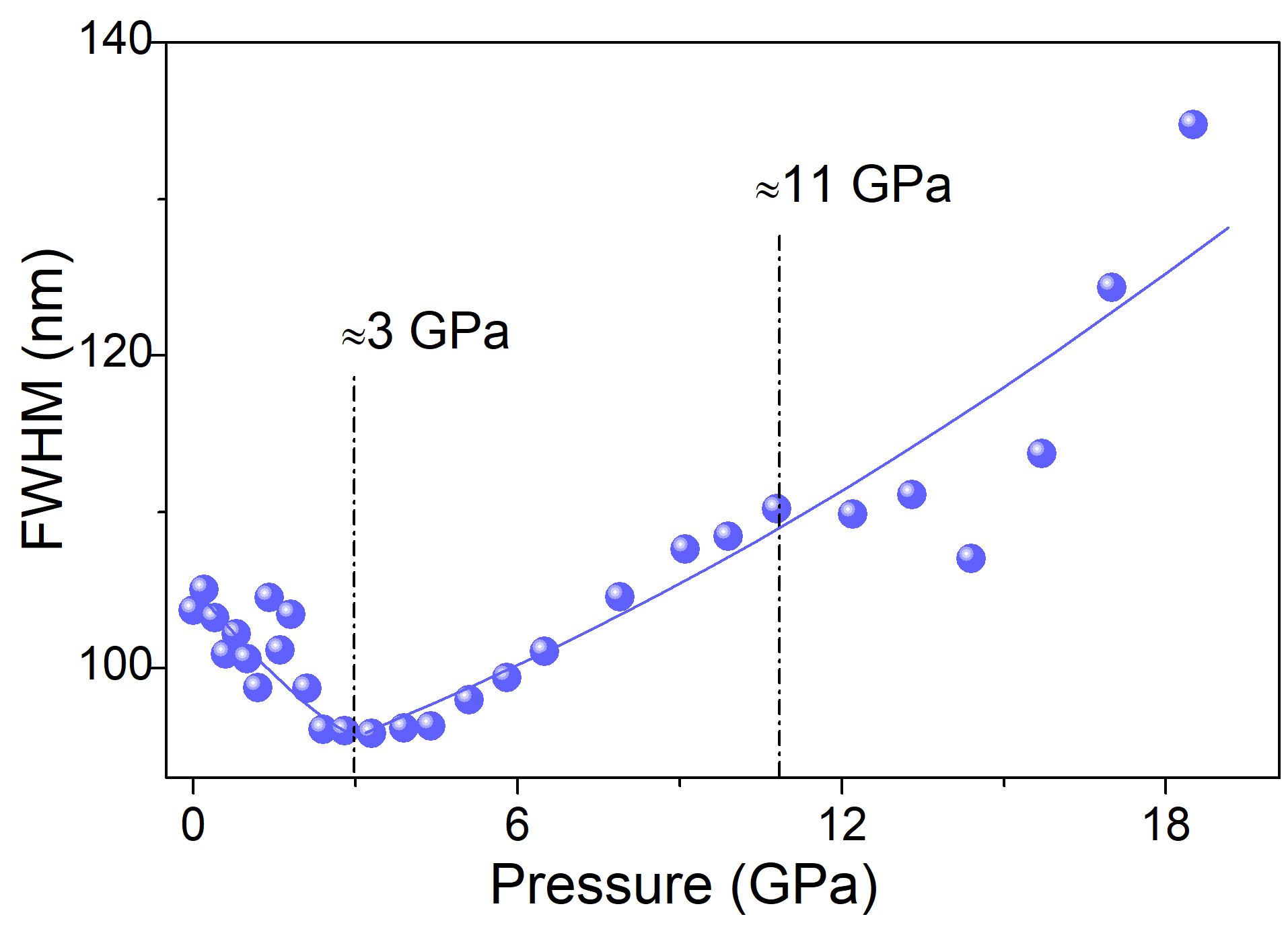
**

**Figure S6.** High-pressure FWHM of the emission spectra of OO.

**
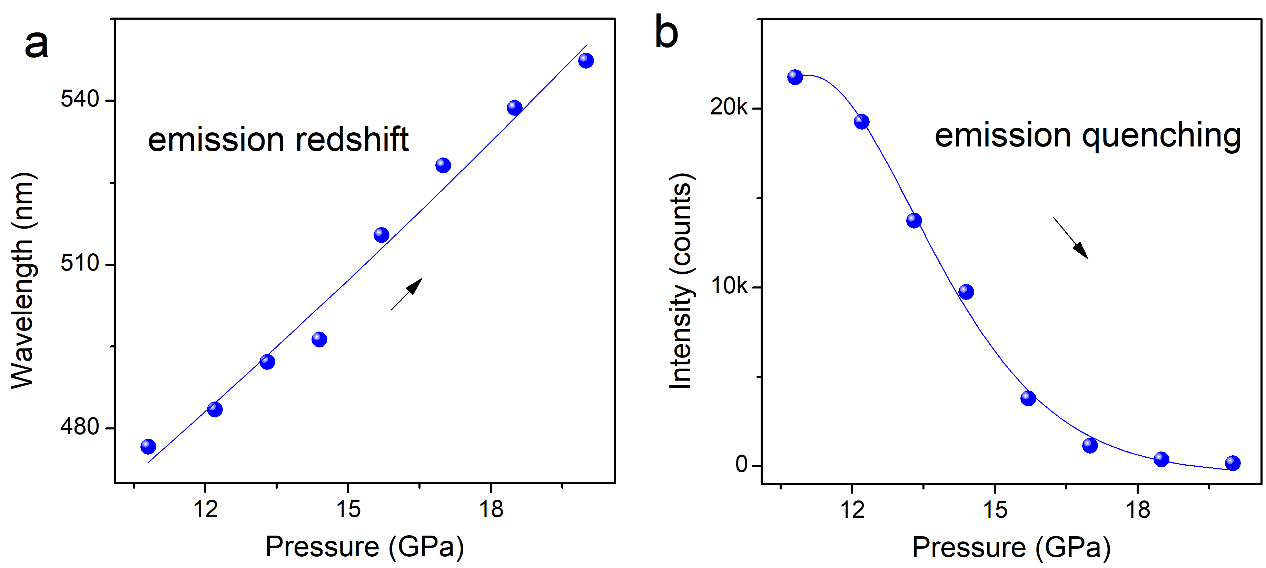
**

**Figure S7.** Pressure-dependent emission (a) wavelength and (b) intensity of OO above ≈11 GPa.

**
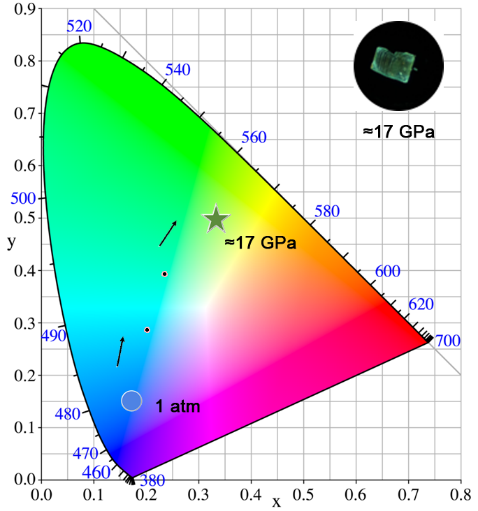
**

**Figure S8.** The typical CIE chromaticity coordinate of OO at selected pressure points. Inset illustrates the PL micrograph of the detected crystal at around 17 GPa.

**
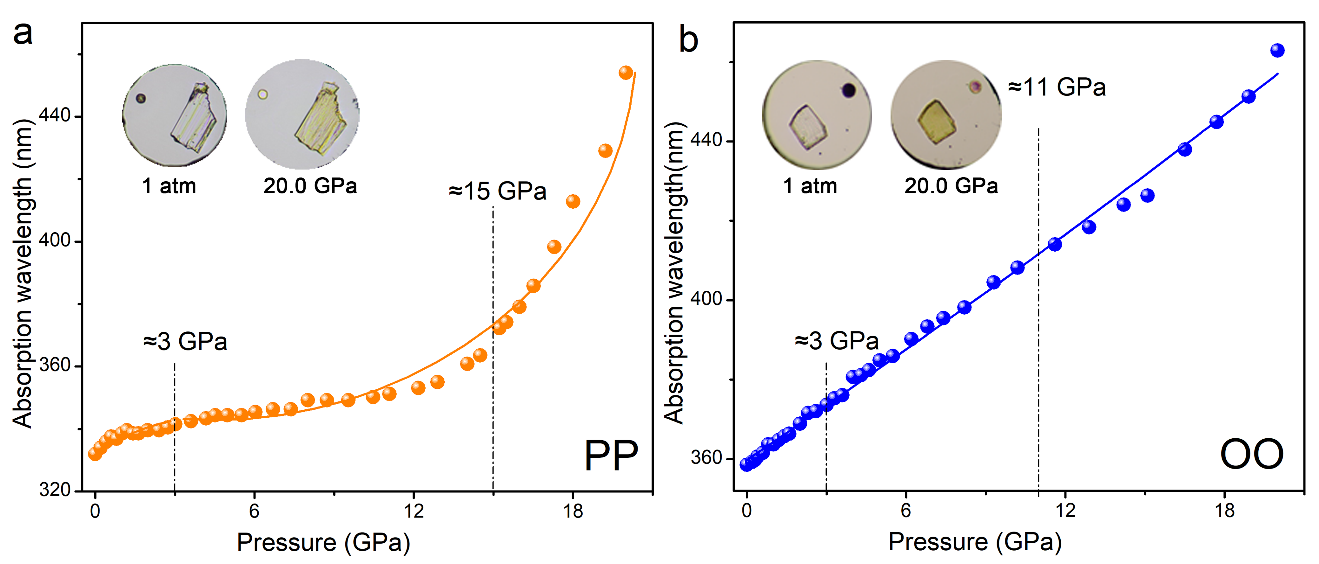
**

**Figure S9.** Pressure-dependent absorption wavelengths of (a) PP and (b) OO crystals, respectively. The insets present the micrographs of the detected crystals under white-light illumination.

**
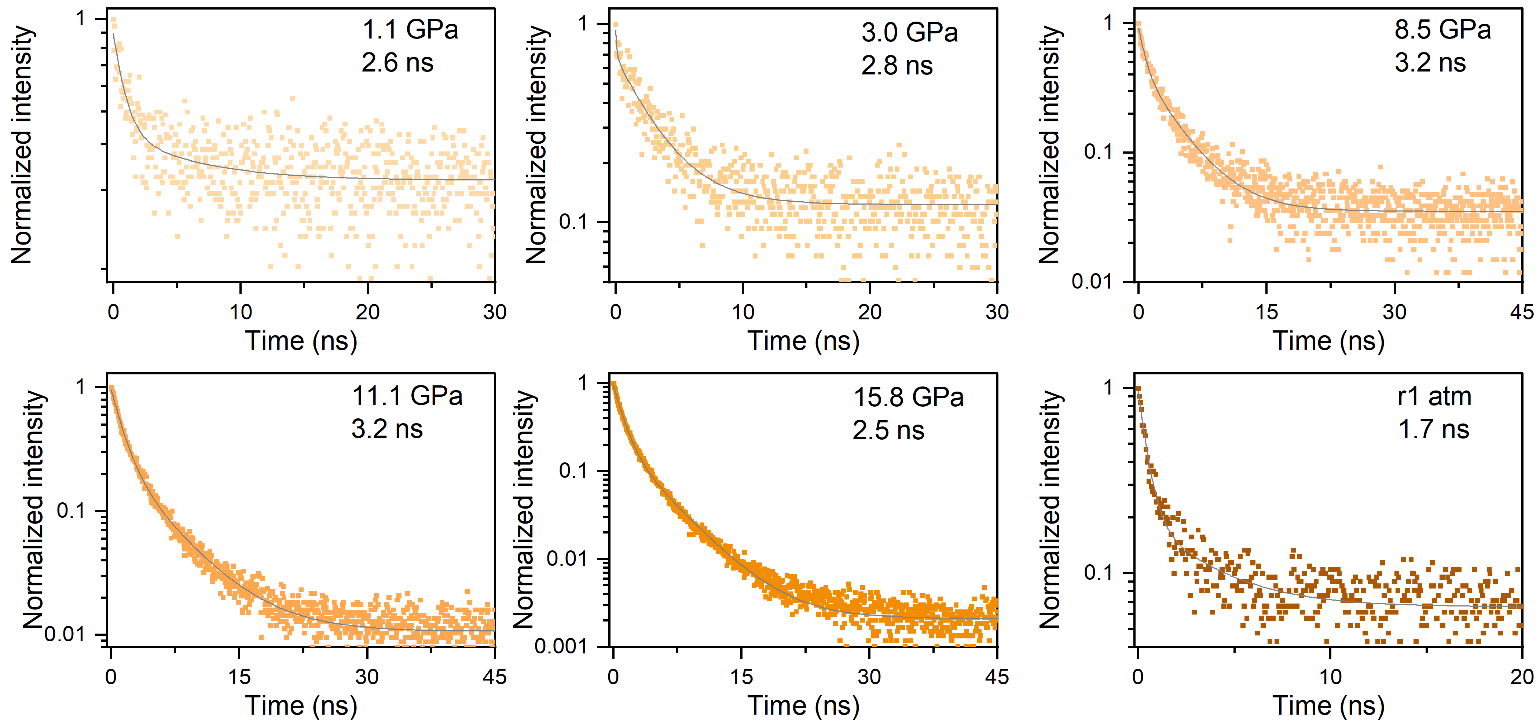
**

**Figure S10.** Selected luminescence decay curves of PP with increasing pressure.

**
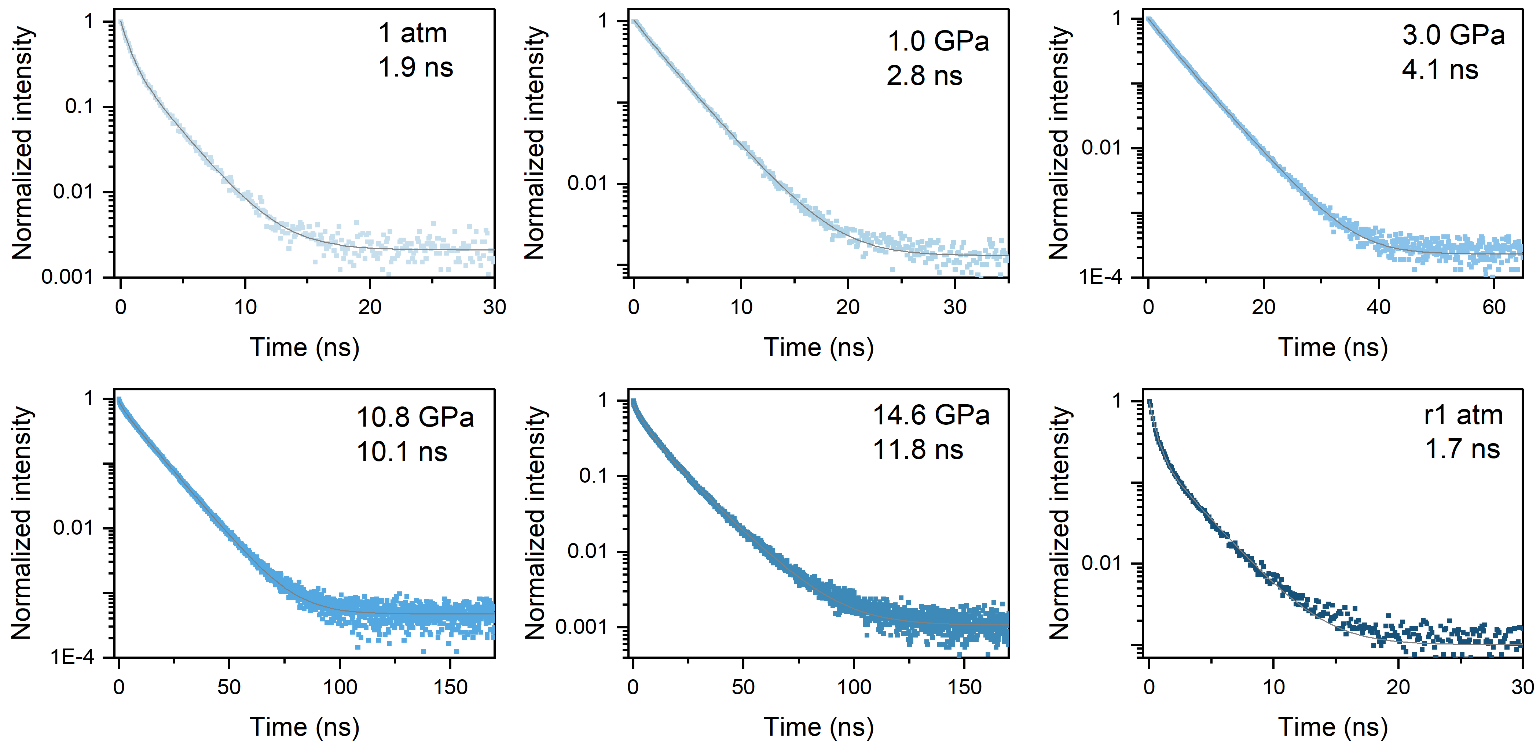
**

**Figure S11.** Typical luminescence decay curves of OO at selected pressure points.

| **Molecule** | **Detected (cm^-1^)** | **Calculated (cm^-1^)** | **Identification** |
| --- | --- | --- | --- |
| **PP** | 119.6 | 98.4 | out-of-plane bending |
|  | 134.2 | 133.7 | out-of-plane bending |
|  | 147.9 | 154.5 | out-of-plane bending |
|  | 194.1 | 206.1 | in-plane distortion |

**Table S3.** Comparisons between the detected and calculated Raman vibrations of PP at 1 atm.

**
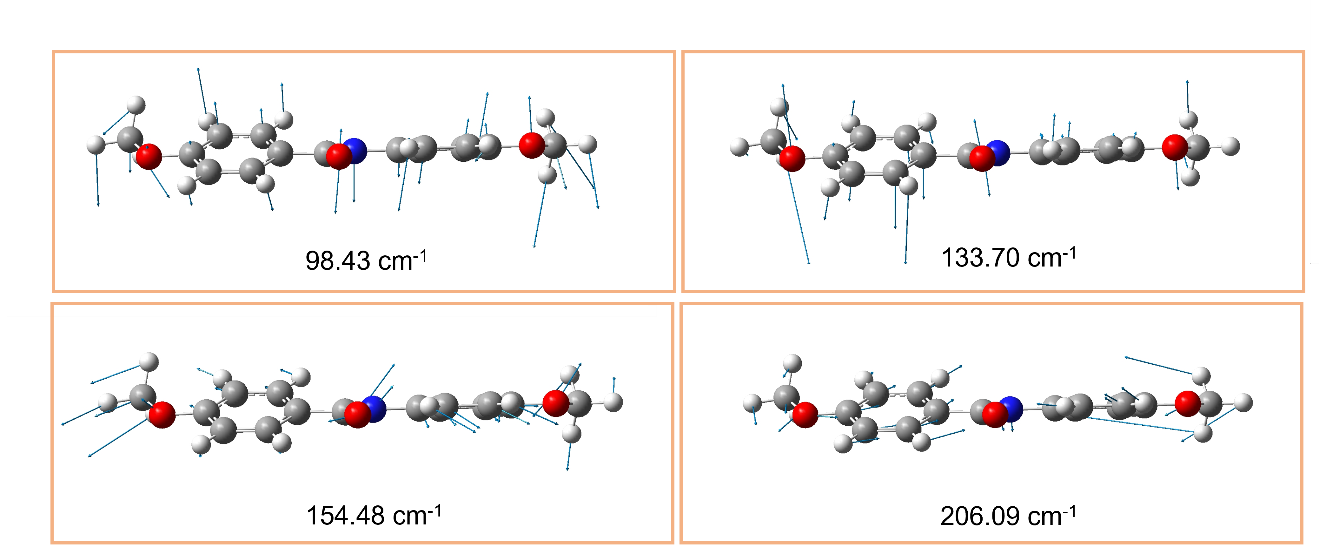
**

**Figure S12.** Illustration of typical low-frequency vibrations of PP. The arrows represent the displacement vectors for the corresponding vibration.

| **Molecule** | **Detected (cm^-1^)** | **Calculated (cm^-1^)** | **Identification** |
| --- | --- | --- | --- |
| **OO** | 93.2 | 85.2 | out-of-plane bending |
|  | 117.6 | 128.0 | out-of-plane bending |
|  | 150.4 | 172.6 | in-plane distortion |
|  | 180.2 | 188.6 | out-of-plane bending |

**Table S4.** Comparisons between the Raman vibrations of OO detected and calculated at 1 atm.

**
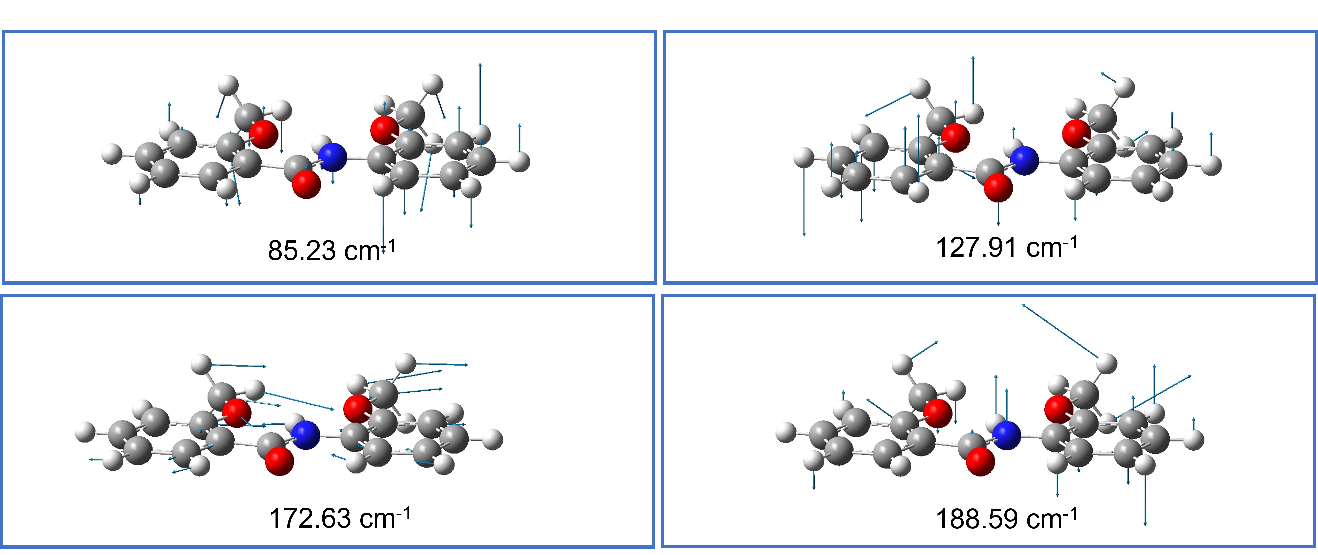
**

**Figure S13.** Illustration of typical low-frequency vibrations of OO. The arrows represent the displacement vectors for the corresponding vibration.

**
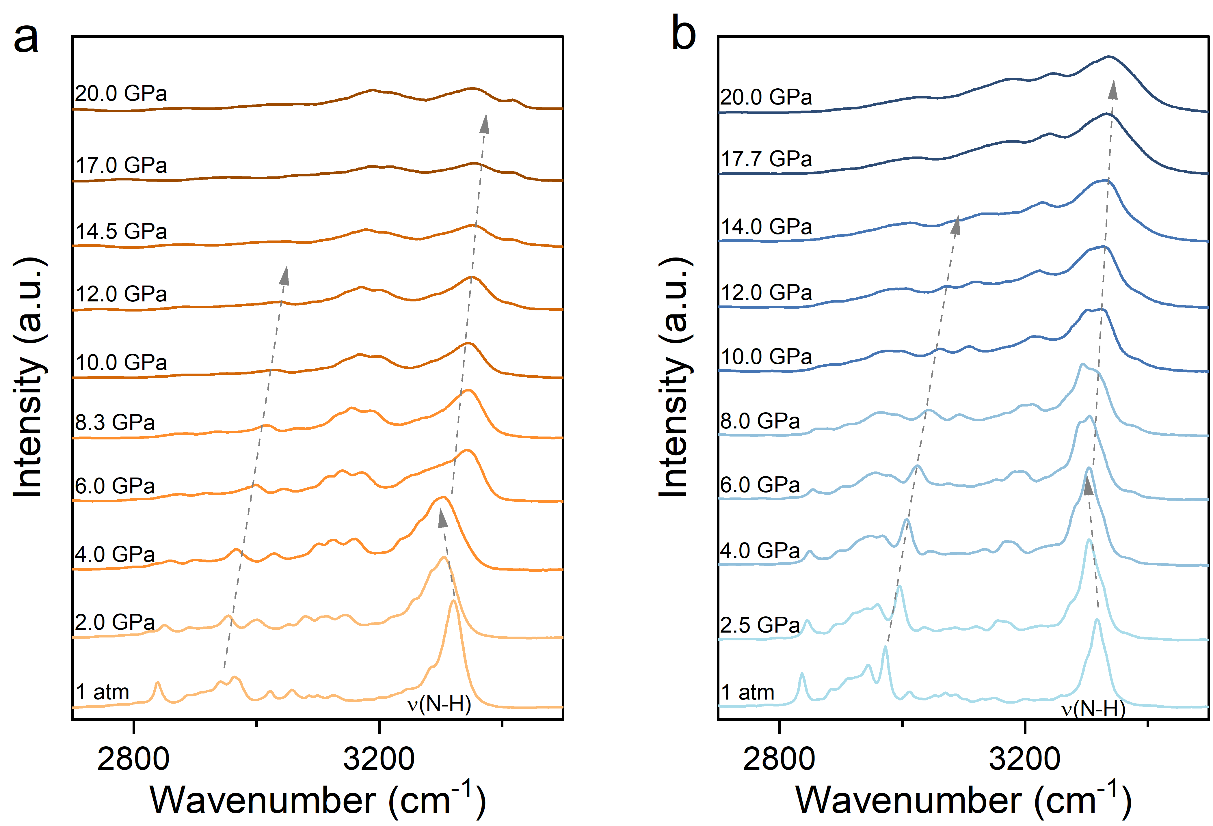
**

**Figure S14.** High-pressure FT-IR spectra between the frequency range of 2700 cm^-1^ – 3500 cm^-1^ of (a) PP and (b) OO, respectively.

**
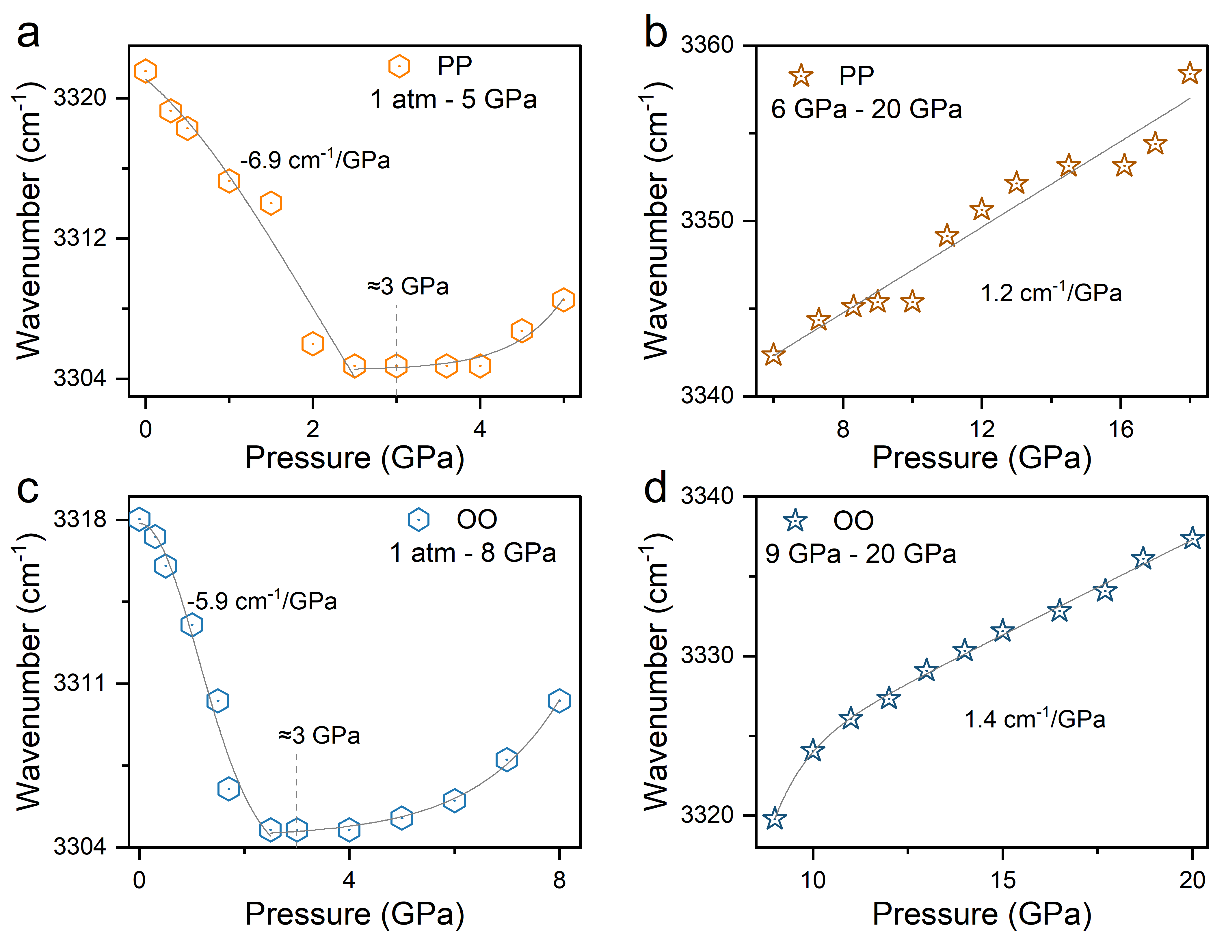
**

**Figure S15.** (a) – (b) Pressure-dependent shifts of typical N-H stretching vibrations of PP in the pressure ranges of (a) 1 atm – 5 GPa and (b) 6 GPa – 20 GPa. (c) – (d) High-pressure shifts of typical N-H vibrations of OO in the pressure ranges of (c) 1 atm – 8 GPa and (d) 9 GPa – 20 GPa.

**
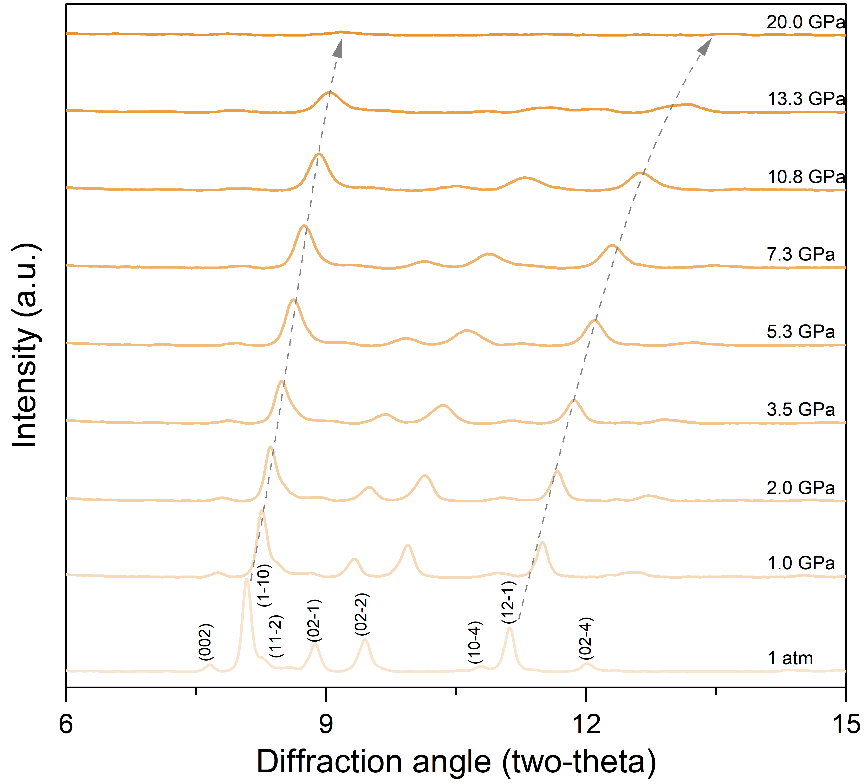
**

**Figure S16.** The selected ADXRD spectra of PP with increasing pressure.

**
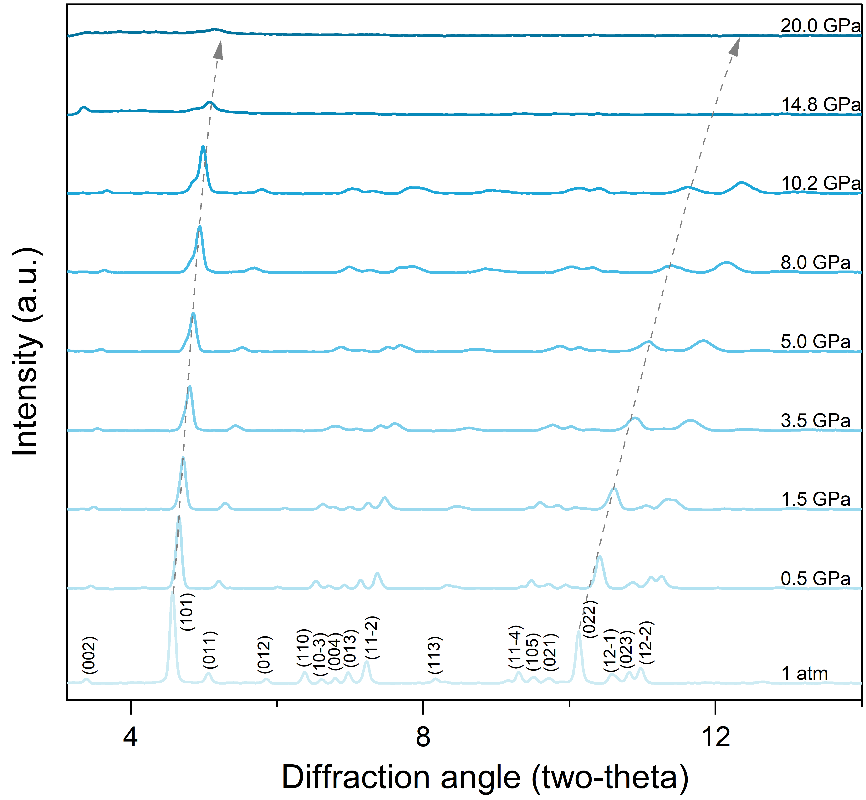
**

**Figure S17.** The selected ADXRD spectra of OO with increasing pressure.

**
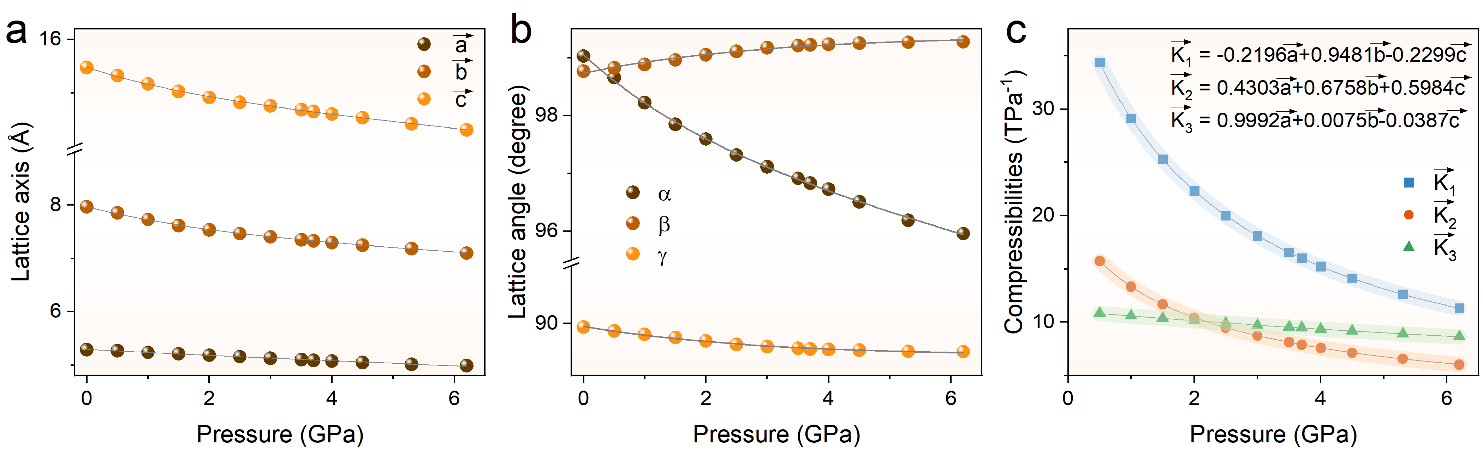
**

**Figure S18.** Pressure-dependent evolution of (a) lattice axes, (b) lattice angle and (c) compressibility of PP.

**
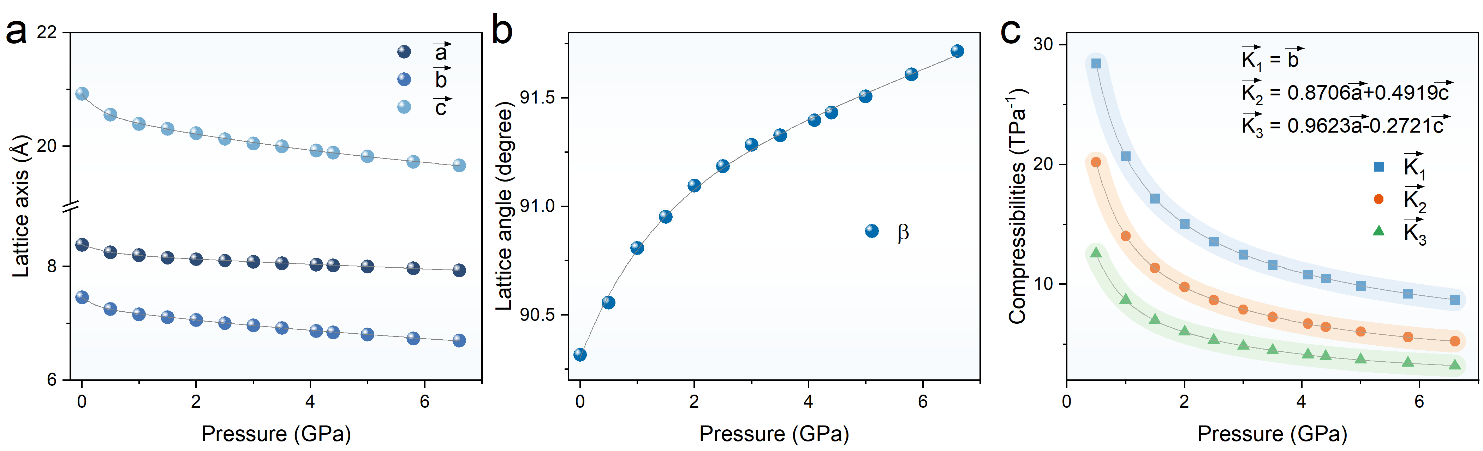
**

**Figure S19.** Pressure-dependent evolution of (a) lattice axes, (b) lattice angle and (c) compressibility of OO.

**
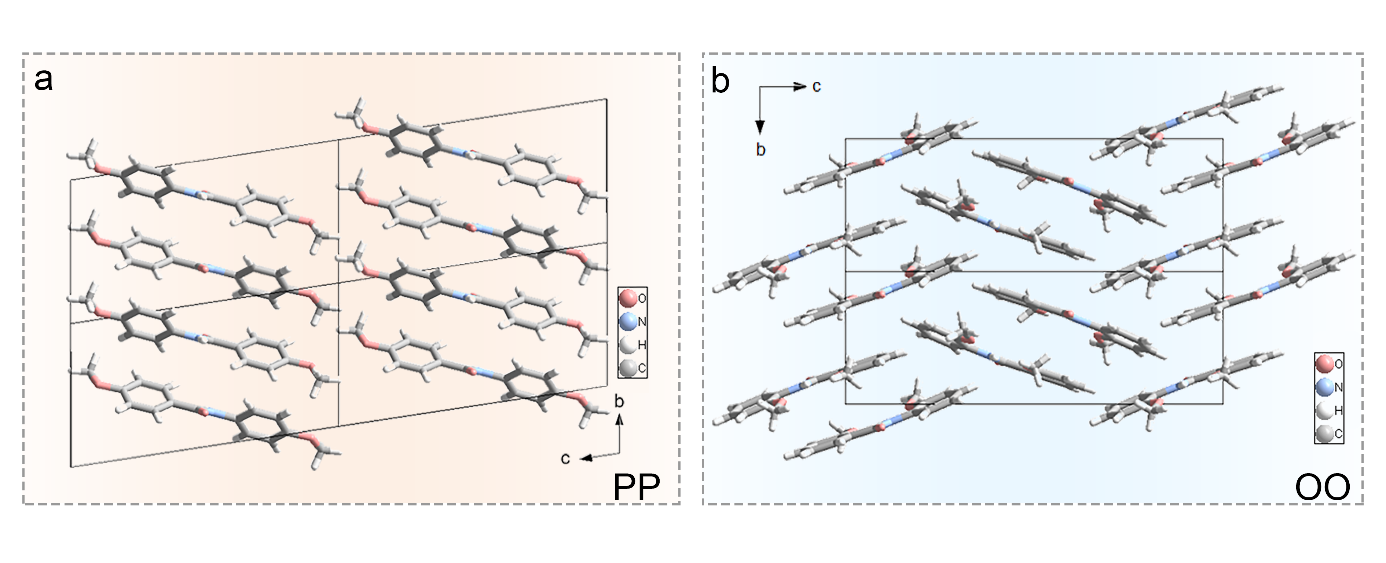
**

**Figure S20**. Structural illustration of the intermolecular stacking along the *b*-axis, (a) PP (b) OO.

**
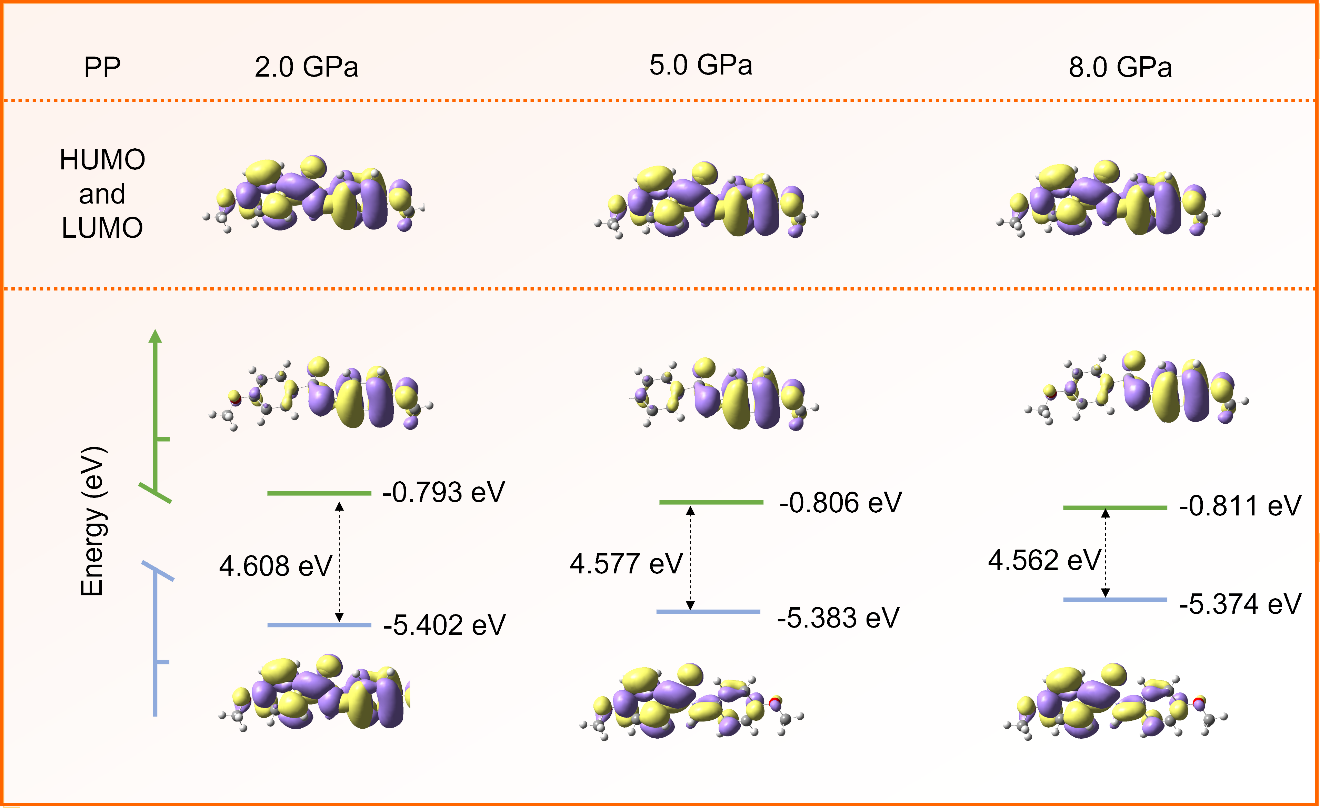
**

**Figure S21.** Frontier molecular orbitals of typical PP molecule calculated at selected pressure points.

**
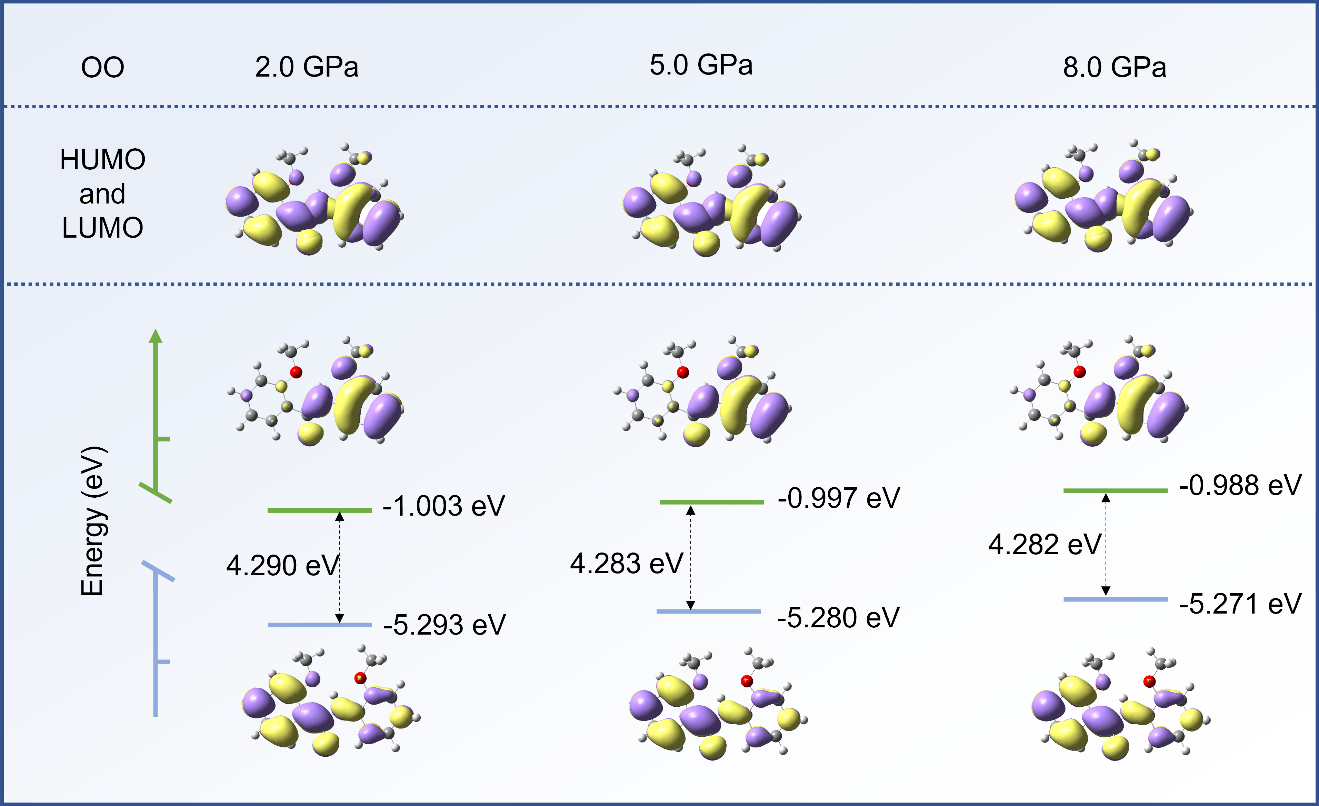
**

**Figure S22.** High-pressure Frontier molecular orbitals of typical OO molecule.


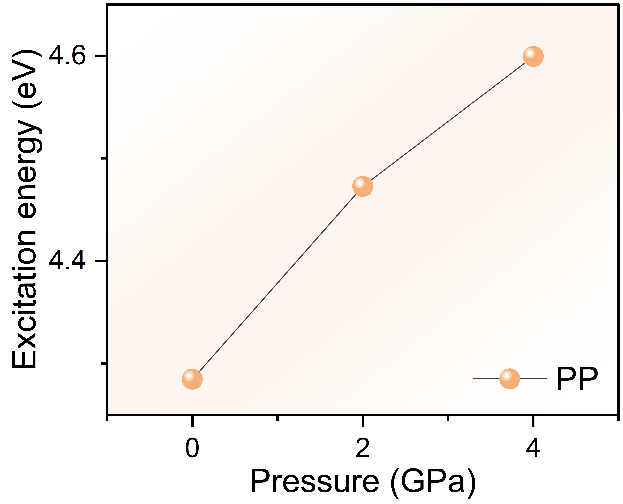


**Figure S23**. The calculated excitation energy of PP molecule with increasing pressure.

**
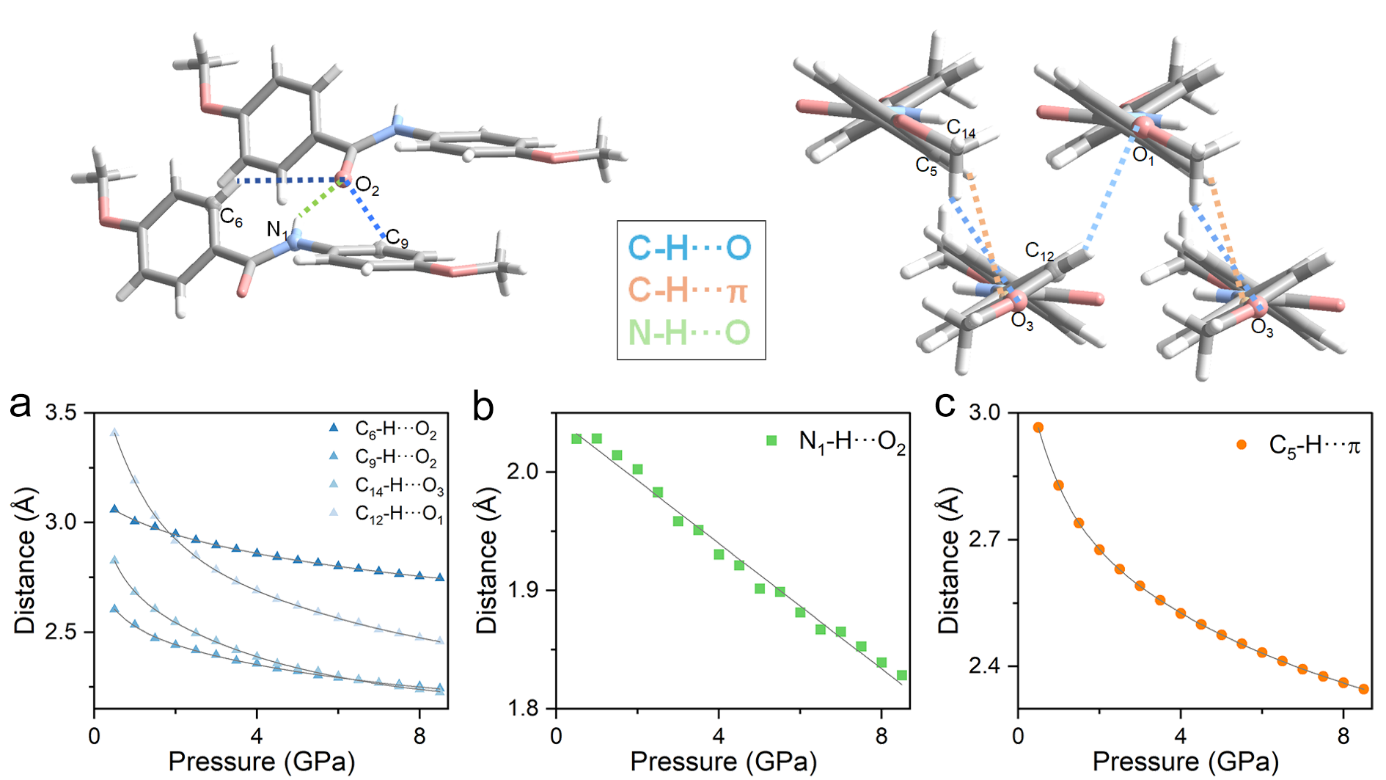
**

**Figure S24.** High-pressure evolution of typical H∙∙∙O and H∙∙∙π distances between the PP molecules.

**
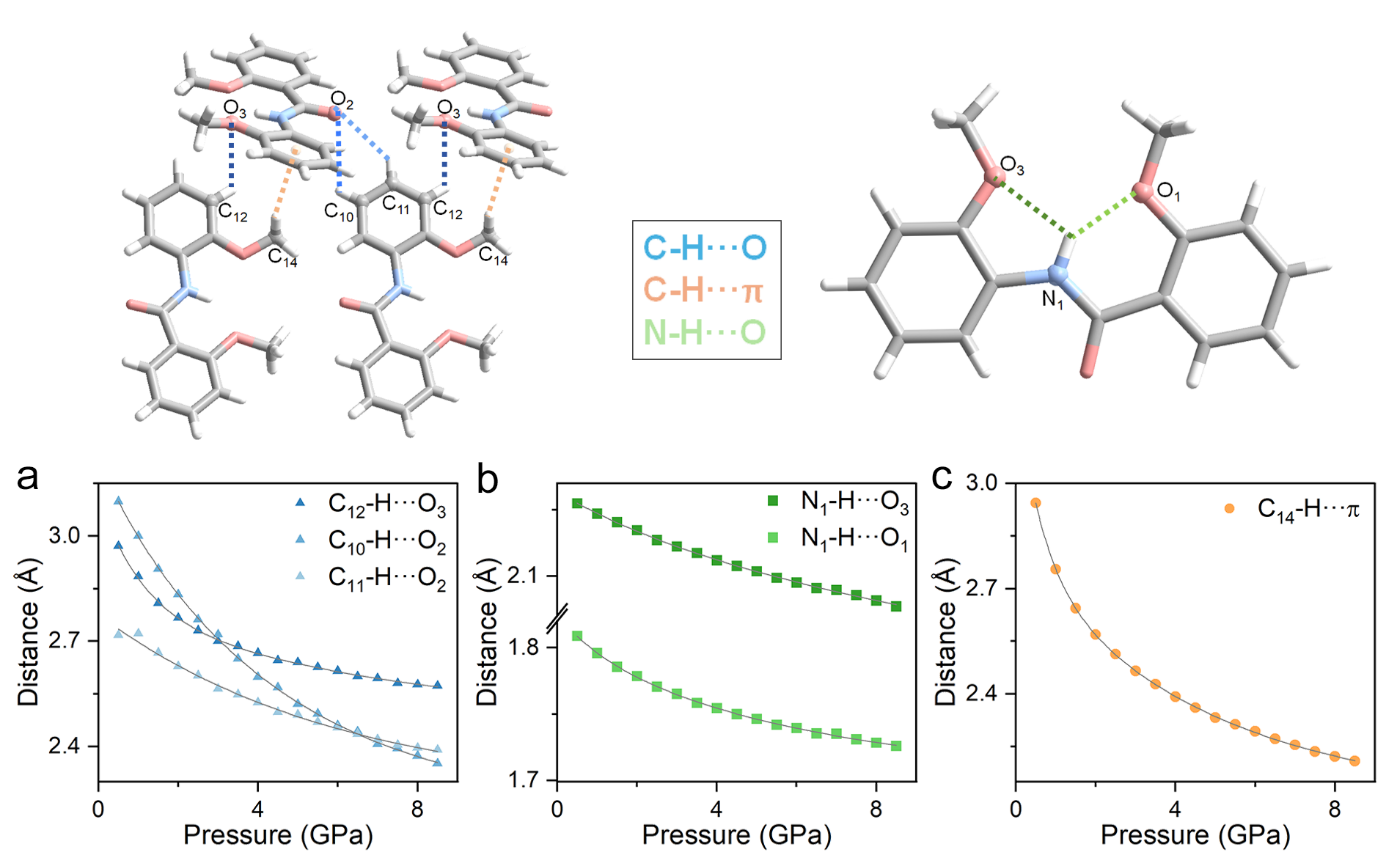
**

**Figure S25.** The pressure-dependent H∙∙∙O and H∙∙∙π distances between the OO molecules with increasing pressure.

**
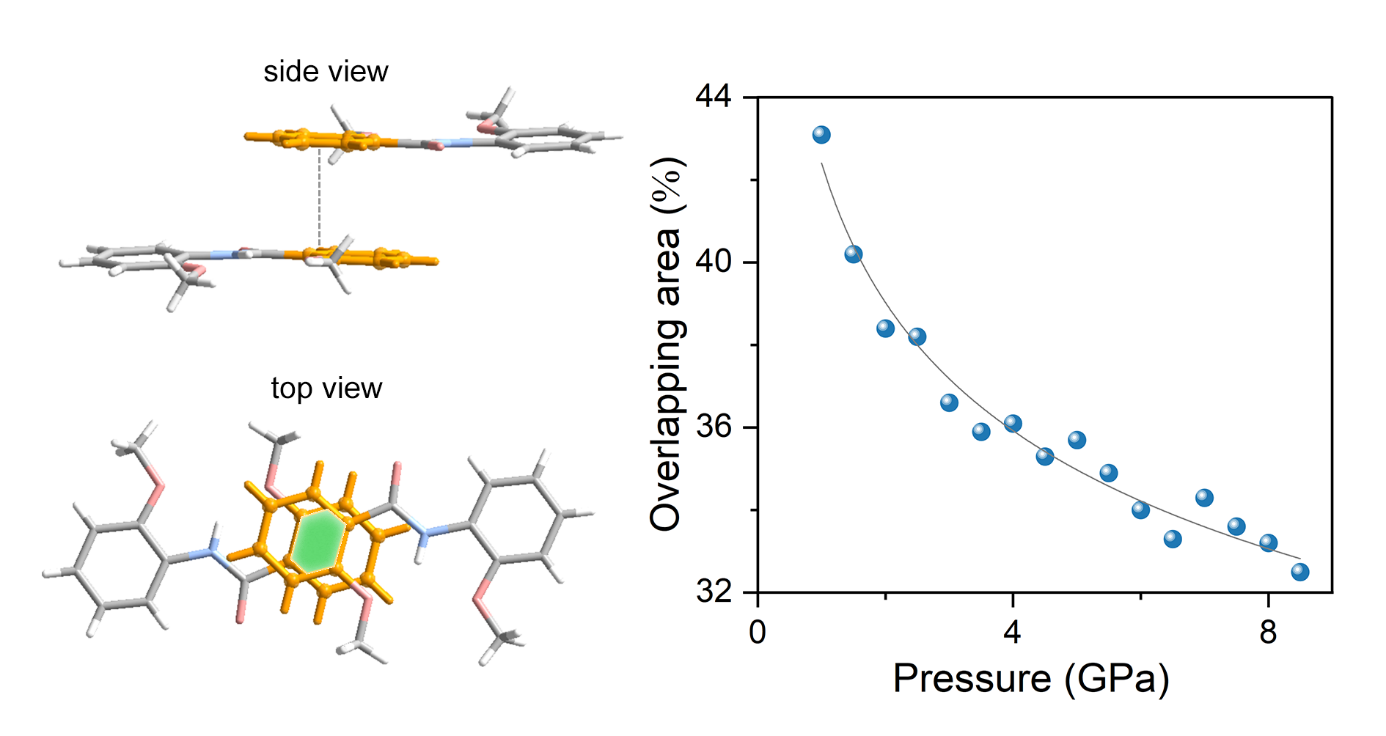
**

**Figure S26.** The pressure-dependent evolution of overlapping area between the neighboring phenyl rings in OO.

**
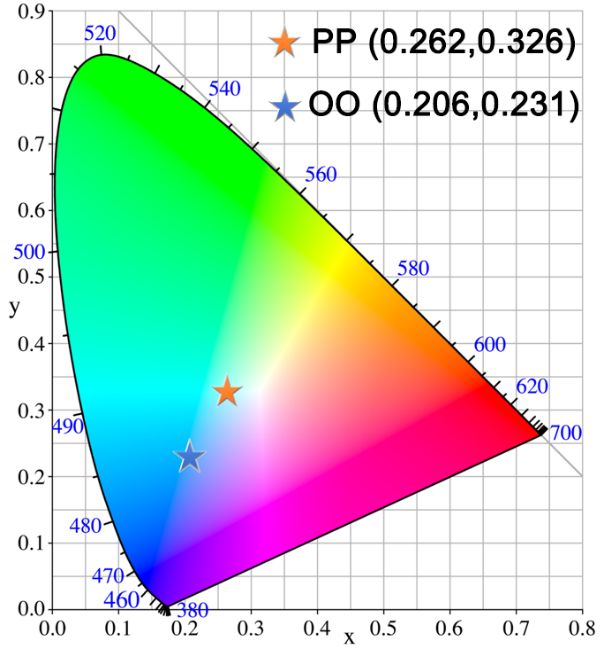
**

**Figure S27.** The CIE chromaticity coordinates of recovered (a) PP and (b) OO after high-pressure treatment of ≈20 GPa.

**
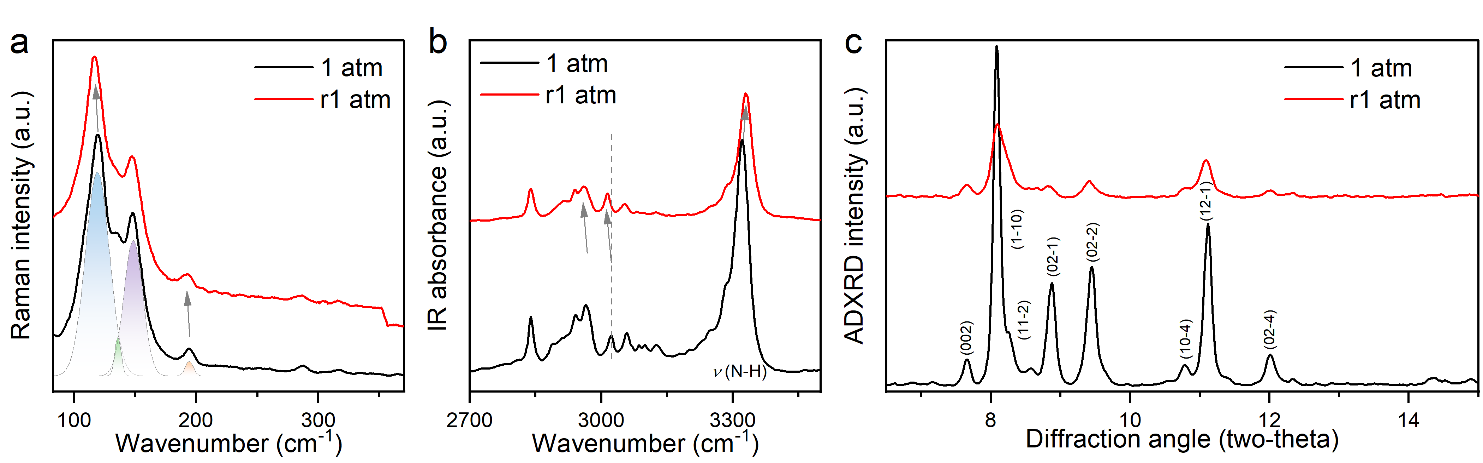
**

**Figure S28.** Comparisons between the (a) Raman spectra, (b) FT-IR spectra, and (c) ADXRD spectra of PP before and after high-pressure treatment of ≈20 GPa.


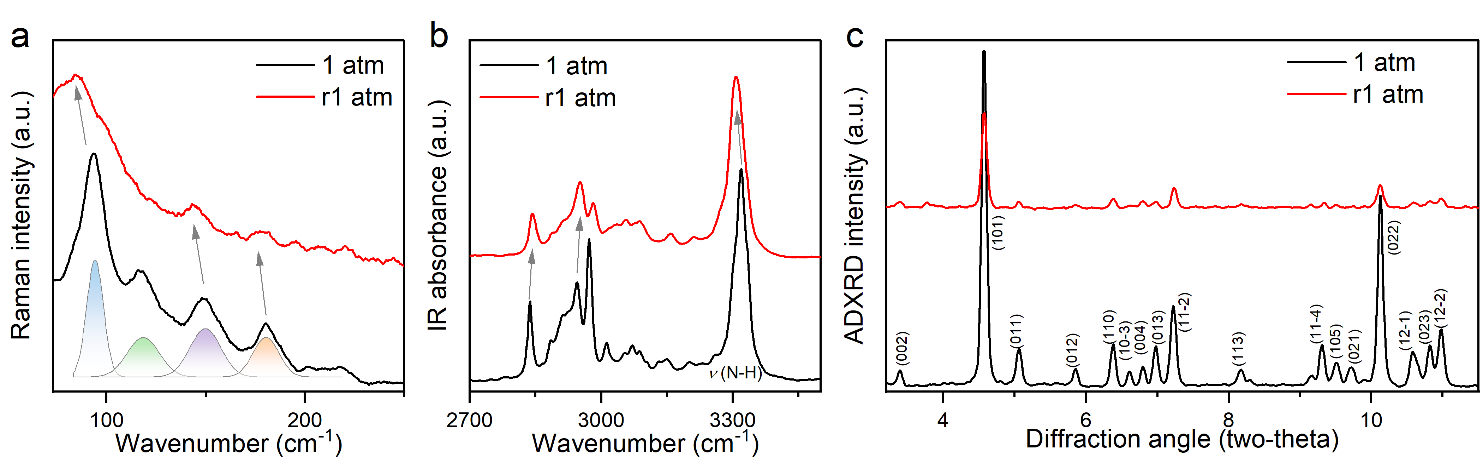


**Figure S29.** Comparisons between the (a) Raman spectra, (b) FT-IR spectra, and (c) ADXRD spectra of OO before and after high-pressure treatment of ≈20 GPa.

**
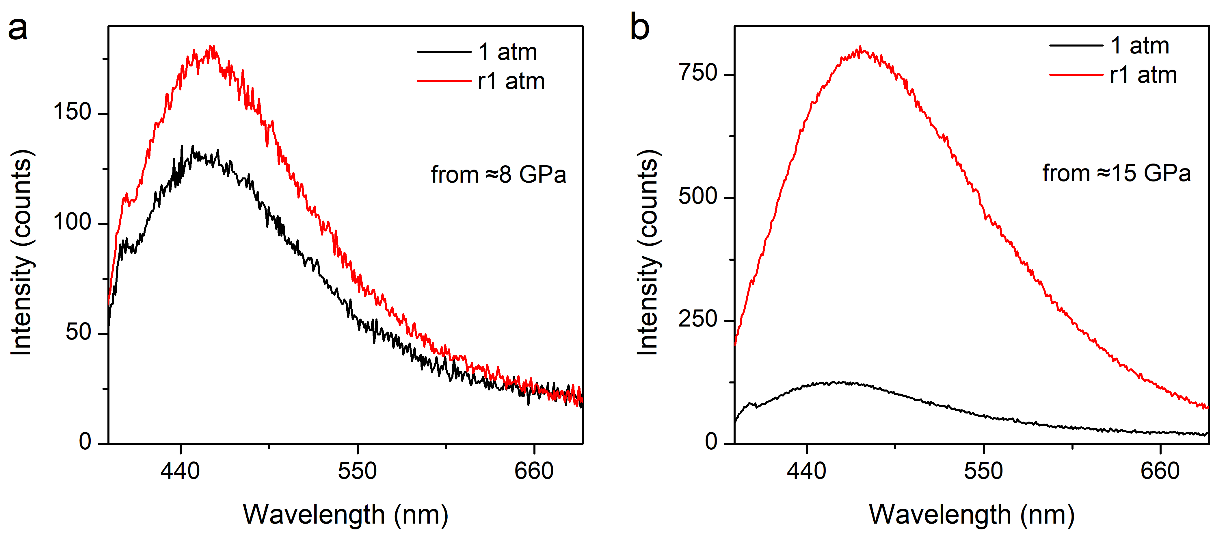
**

**Figure S30.** Comparisons between the emission spectra of PP before and after pressure treatment of (a) from ≈8 GPa and (b) from ≈15 GPa. The critical pressures are chosen based on the pressure range of emission enhancement phenomenon.

**
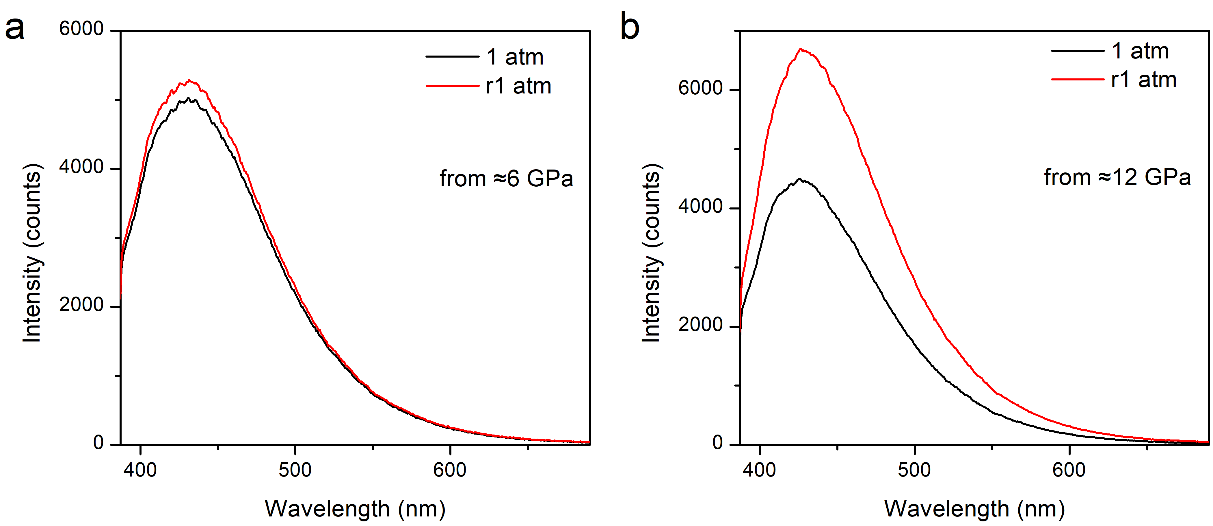
**

**Figure S31.** Comparisons between the emission spectra of OO before and after pressure treatment of (a) from ≈6 GPa and (b) from ≈12 GPa. The critical pressures are chosen based on the pressure range of emission enhancement phenomenon.


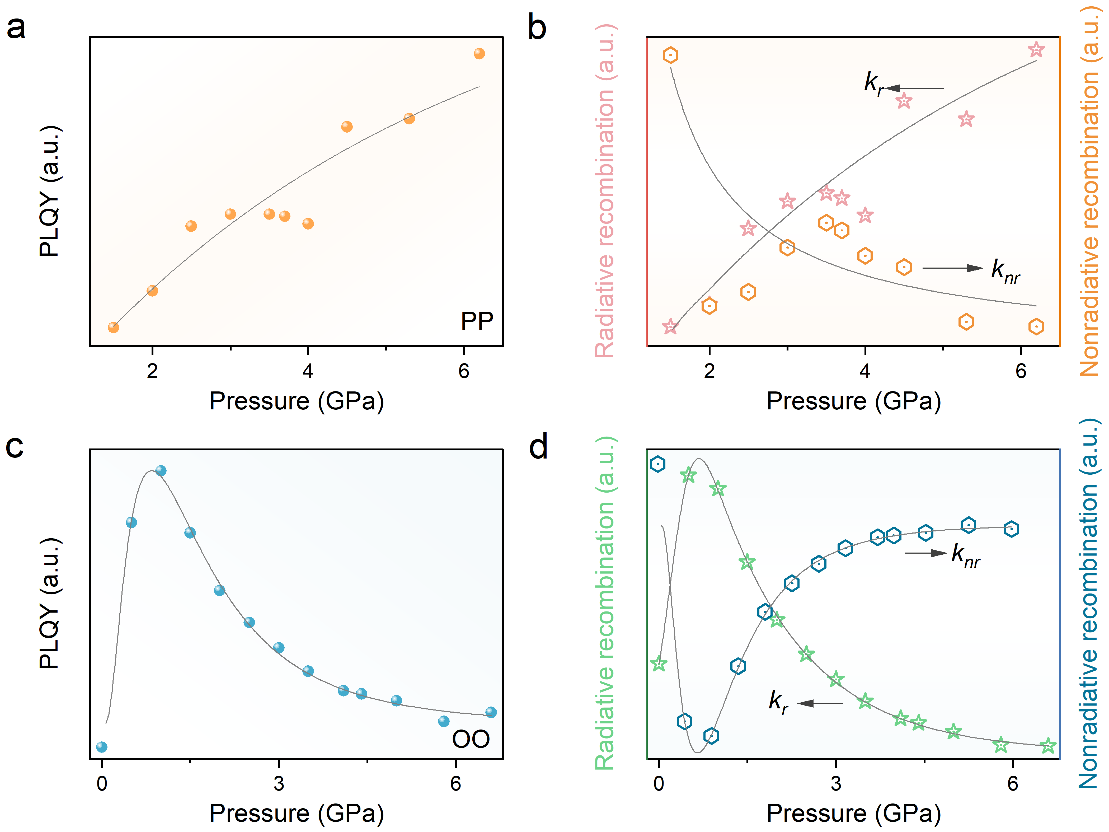


**Figure S32**. Pressure-dependent evolution of photoluminescence quantum yield (PLQY) of (a) PP and (c) OO as well as radiative rate (*k_r_*) and nonradiative rate (*k_nr_*) of (b) PP and (d) OO.

The calculated PLQY of PP keeps gradually increases upon compression, which is coincident with the emission enhancement. The dissentious evolution around 3 GPa in also observed in luminescence experiments. Meanwhile, the *k_r_* also exhibits gradually increases upon compression, accompanied with the basically unchanged *k_nr_*. This phenomenon is coincident with the mechanism inferred in lifetime and Stokes shift.

As for the OO molecule, the initial compression increases the PLQY with emission enhancement. This phenomenon is associated with the increased *k_r_* and decreased *k_nr_*, which is coincident with the basically unchanged luminescence lifetime. Meanwhile, the further significant increased *k_nr_* with further compression should account for the emission quenching.

***Reference***

[1] M. Lian, Y. Mu, Z. Ye, Z. Lu, J. Xiao, J. Zhang, S. Ji, H. Zhang, Y. Huo, B. Z. Tang, “Manipulating noncovalent conformational lock via side-chain engineering for luminescence at aggregate level” *Aggregate* **2024**, *5*, e560.

[2] G. Barbe, A. B. Charette, “Highly Chemoselective Metal-Free Reduction of Tertiary Amides” *J. Am. Chem. Soc.* **2008**, *130*, 18-19.

[3] T. Ben Halima, J. K. Vandavasi, M. Shkoor, S. G. Newman, “A Cross-Coupling Approach to Amide Bond Formation from Esters” *ACS Catalysis* **2017**, *7*, 2176-2180.

[4] P. Kannan, T. S. Rao, N. Rajendran, “Improvement in the Corrosion Resistance of Carbon Steel in Acidic Condition Using Naphthalen-2-ylnaphthalene-2-carboxammide Inhibitor” *J Colloid Interface Sci* **2018**, *512*, 618-628.

[5] A. W. Lawson, T.-Y. Tang, “A Diamond Bomb for Obtaining Powder Pictures at High Pressures” *Rev. Sci. Instrum.* **1950**, *21*, 815.

[6] J. C. Chervin, B. Canny, M. Mancinelli, “Ruby-spheres as pressure gauge for optically transparent high pressure cells” *High Pressure Res.* **2001**, *21*, 305-314.

[7] C. Prescher, V. B. Prakapenka, “DIOPTAS: a program for reduction of two-dimensional X-ray diffraction data and data exploration” *High Pressure Res.* **2015**, *35*, 223-230.

[8] J. S. Wilson, N. Chawdhury, M. R. A. Al-Mandhary, M. Younus, M. S. Khan, P. R. Raithby, A. Köhler, R. H. Friend, “The Energy Gap Law for Triplet States in Pt-Containing Conjugated Polymers and Monomers” *J. Am. Chem. Soc.* **2001**, *123*, 9412-9417.

[9] Y. Wang, S. Guo, H. Luo, C. Zhou, H. Lin, X. Ma, Q. Hu, M.-h. Du, B. Ma, W. Yang, X. Lü, “Reaching 90% Photoluminescence Quantum Yield in One-Dimensional Metal Halide C_4_N_2_H_14_PbBr_4_ by Pressure-Suppressed Nonradiative Loss” *J. Am. Chem. Soc.* **2020**, *142*, 16001-16006.
